# Toward an Understanding of the Psychological Meaning of Physiological Synchrony

**DOI:** 10.1111/psyp.70404

**Published:** 2026-09-25

**Authors:** Chad Danyluck, Nissa Doyle, Tyler Thorne, Andy Adler

**Affiliations:** ^1^ Carleton University Ottawa Ontario Canada

**Keywords:** autonomic nervous system, interpersonal physiology, physiological synchrony, psychophysiological inference, theoretical review

## Abstract

Physiological synchrony—coordinated changes in physiological activity between individuals—is a frequently studied yet variably interpreted phenomenon in psychological science. Decades of research show that physiological synchrony is ubiquitous across relationship and social contexts, but consensus remains elusive about what it means psychologically. We advance an inferential framework for mapping observed synchrony to warranted psychological conclusions by importing classical principles of psychophysiological inference into the interpersonal domain. Building on Cacioppo and Tassinary's logic of inference, we treat physiological synchrony as an empirical pattern whose meaning depends on three constraints: specificity (which psychological processes remain plausible given the physiological pattern and plausible alternatives), generality (the contexts, relationships, and timescales over which an interpretation should hold), and sensitivity (detectability of the theoretically relevant dynamics given measurement and modeling choices). We ground these constraints in principles of autonomic organization, emphasizing how physiological ambiguity and modes of control shape what synchrony can and cannot mean. We use established approaches to pattern‐based psychophysiological inference, including autonomic modes (i.e., configurations of sympathetic–parasympathetic control) and the biopsychosocial model of challenge and threat, to illustrate how multivariate physiological profiles and theoretically relevant contextual information can constrain psychological interpretation. We further argue that model class is an inferential commitment: choices about sampling rate, observation window, preprocessing, and whether coupling is modeled as contemporaneous, lagged, or dynamical determine which forms of coordination are detectable and which interpersonal processes remain viable candidates for interpretation. By integrating contemporary synchrony approaches within this inferential framework, we provide conceptual and methodological guidance for clarifying when physiological synchrony supports strong psychological inference and for specifying the boundary conditions under which those interpretations should generalize.

## Impact Statement

Physiological synchrony is widely documented across relationships and contexts, yet its psychological interpretation is often inferred without an explicit inferential framework. We advance the field by adapting the logic of classical psychophysiological inference to the interpersonal domain, specifying how specificity, generality, and sensitivity constrain what physiological synchrony estimates can—and cannot—support. This framework makes interpretations of physiological synchrony more bounded and comparable across studies, providing a foundation for a more cumulative science of interpersonal physiology.

Across diverse areas of psychological science, researchers have become captivated by the observation that social partners' physiological systems often synchronize. Caregivers' and infants' heart rhythms align during attuned interactions (Feldman et al. 2011), romantic partners' pupils dilate in unison while viewing emotionally charged scenes (Kang and Wheatley 2017), teammates' electrodermal activity covaries in moments of collective tension (Mønster et al. 2016), and strangers' sympathetic and parasympathetic responses become coordinated as friendship emerges (Danyluck and Page‐Gould 2018, 2019). The ubiquity and intuitive appeal of physiological synchrony have made it a compelling phenomenon for understanding how social experiences become embodied across people.

Yet its psychological meaning remains unclear: physiological synchrony has been interpreted as reflecting emotion contagion, shared attention, stress transmission, co‐regulation, social bonding, and other interpersonal processes (Palumbo et al. 2017). Although this diversity of interpretation is scientifically generative, it has also created a conceptual impasse. Without a principled framework for linking observed physiological synchrony to underlying psychological processes, findings risk being over‐interpreted, under‐specified, or simply incomparable across studies.

We argue that this ambiguity reflects a missing theoretical layer: a framework for psychophysiological inference at the interpersonal level—that is, for reasoning from observed physiological patterns to psychological meaning. Classical psychophysiology has long emphasized that physiological signals do not map directly onto psychological states but instead acquire meaning through disciplined inference (Cacioppo and Tassinary 1990). Yet these inferential principles have rarely been applied systematically to the study of physiological synchrony. Without such a framework, decisions about what to measure, how to model synchrony, and how to interpret its presence are made largely in isolation from the inferential goals they are meant to serve.

The current paper addresses this gap by advancing a framework for specifying when, and under what inferential constraints, researchers can make claims about the *psychological meaning* of physiological synchrony.^1^ Because this framework applies classical principles of psychophysiological inference to an expanding physiological synchrony literature marked by renewed and broadened interest across methodological traditions, we retain precise psychophysiological terminology while defining key terms on first use and using tables, figures, and explanatory boxes throughout.^2^

We argue that the interpretive challenges facing the field stem from insufficient attention paid to two key inferential dimensions: specificity—how narrowly a physiological observation limits the range of plausible psychological interpretations—and generality—when, where, and under what conditions those interpretations are warranted. Drawing from classical psychophysiological theory (Cacioppo and Tassinary 1990), we show how these dimensions can be applied to interpersonal physiological data and integrated with contemporary models of physiological synchrony (Feldman 2012; Gordon et al. 2024; West and Mendes 2023). Consistent with Cacioppo and Tassinary, we argue that these inferential dimensions can only be meaningfully evaluated when the physiological measures and analytic approaches used are sufficiently sensitive to detect the temporal dynamics of the psychological processes under study.

Reintroducing this inferential logic reorients physiological synchrony research toward the fundamental question that motivated psychophysiology from the start: *What can physiological signals tell us about the mind?* By applying this question to the interpersonal level, we can move beyond demonstrating that physiological synchrony exists as a phenomenon worth studying toward clarifying when, how, and why physiological synchrony carries *psychological meaning*.

## 1 Historical Background: Seven Decades of Intermittent Discovery

It has been seventy years since the first empirical study of physiological synchrony was published (Di Mascio et al. 1955). The study was originally designed to evaluate a set of tools for assessing psychotherapeutic interviews and to introduce “sociophysiology” as a new interdisciplinary domain linking social behavior and physiological arousal. It demonstrated that psychiatrists' and patients' heart rates often fluctuated together across time. This finding was tentatively interpreted as evidence that the presence of concordant or in‐phase synchrony might be a marker of therapeutic rapport (Di Mascio et al. 1955). Over the next decade, two additional studies of physiological synchrony appeared, again in clinical contexts, offering further glimpses of coordinated physiological arousal between people (Coleman et al. 1956; Kaplan et al. 1963). Then, for almost two decades, the topic largely receded from academic inquiry.

The early 1980s brought a modest resurgence of interest in physiological synchrony alongside more explicit attempts to describe its psychological meaning. In counseling dyads, Robinson et al. (1982) treated synchrony as a marker of empathy. They argued that rapid matching of phasic skin‐conductance responses should track affective matching and showed that this correspondence correlated with clients' ratings of empathic understanding. Soon after, interest expanded into new social contexts. A highly influential study demonstrated that dissatisfied romantic couples display greater physiological synchrony during conflict than satisfied couples (Levenson and Gottman 1983). Shortly thereafter, researchers began documenting synchrony in parent–infant interactions (Field et al. 1989), within peer networks (Goldstein et al. 1989), and between strangers (Levenson and Ruef 1992).

Together, these studies provided early evidence that physiological synchrony may serve important social functions, with many explicitly framing physiological synchrony as reflecting empathy or emotional attunement, extending earlier ideas about therapeutic rapport. Yet these interpretations were not grounded in an explicit framework for drawing psychophysiological inferences: they often assessed empathy/attunement in ways that captured only a subset of its components—typically brief ratings (e.g., clients' ratings of empathic understanding; Robinson et al. 1982) or concordance‐based proxies (e.g., inferring attunement from behavioral and physiological concordance in the absence of a direct empathy measure; Field et al. 1989; Goldstein et al. 1989), and were not embedded in a unified theory of interpersonal physiology. In the absence of such a framework, this second wave of work ultimately did more to establish that physiological synchrony occurs across diverse relationship and social contexts than to explain what it signifies psychologically. As a result, despite their pioneering work, these early studies were not taken up as the basis for a broader, unified theory of physiological synchrony.

In the decades since, interest in physiological synchrony has expanded dramatically—spurred by inexpensive technology and by advances in dyadic, group‐level, and time‐series analysis that make it possible to quantify dynamic coupling between individuals in real time. This third wave of research documents physiological synchrony across an even wider range of physiological systems, as well as social and relationship contexts. Yet the field continues to struggle to articulate the psychological significance of physiological synchrony. Researchers have proposed myriad interpretations—affect contagion or stress transmission (Waters et al. 2014; West and Mendes 2023), co‐regulation (Abney et al. 2021; Coutinho et al. 2020), empathy (Levenson and Ruef 1992), shared attention (Filho et al. 2017), and social presence (Ekman et al. 2011). Although some studies provide stronger inferential scaffolding than others (e.g., Waters et al. 2014), these claims are rarely tied explicitly to a broader framework specifying the constraints that delimit psychological interpretation. The central problem, we argue, is that although contemporary studies of physiological synchrony are often motivated by psychological constructs, discussions typically provide limited guidance for how particular measurement and modeling choices constrain the psychological inferences that can be drawn from the observed synchronization of physiological signals. Before treating physiological synchrony as evidence for any specific interpersonal process, researchers must first clarify how physiological patterns can be used to make principled inferences about the mind.

## 2 The Missing Foundation: Psychophysiological Inference

The central difficulty in interpreting physiological synchrony is not simply a lack of data or analytic sophistication, but the absence of a guiding framework for psychophysiological inference—that is, for reasoning from physiological observations to psychological meaning. Curiously, as interest in physiological synchrony was re‐emerging in the 1980s and 1990s, substantial theoretical ground was being broken elsewhere on precisely how to make inferences about psychological states from physiological signals. Cacioppo and Tassinary (1990) were developing a framework of psychophysiological inference, articulating how psychologists could reason from patterns of physiological activity to the mental states that produce them (Table 1). Although this theoretical work was developed primarily to guide inferences about *intra*personal phenomena, it provides the conceptual foundation urgently needed for drawing inferences about *inter*personal phenomena such as physiological synchrony. The next section revisits these classical principles, illustrating how they can guide a new generation of theory‐driven research on the psychological meaning of physiological synchrony.

**TABLE 1:** Inferential framework for psychophysiological research: key terms and phrases in plain language.

Key inferential terms		Plain language		
Psychophysiological inference		Using physiological observations to make cautious claims about psychological processes, ideally guided by theory.		
Specificity		How much a physiological observation or pattern narrows the range of plausible psychological interpretations. For example, heart rate alone may be compatible with many states, whereas a theory‐specified pattern across measures may make some interpretations more plausible than others.		
Generality		When, where, and under what conditions a psychological interpretation of a physiological observation is warranted.		
Sensitivity		Whether the measures and analyses are capable of detecting the expected bodily change at the right time, given the quality and timing of the data—for example, a brief rise in skin conductance during a stressful moment, physiological recovery after a conflict, or delayed linkage between one partner's response and the other's.		
Inferential Constraint		A theoretical, design, measurement, or analytic feature that narrows the psychological interpretations warranted by physiological observations.		
Inferential Commitment		A design or analysis choice, such as which measures, model class, or time window is used, that defines what a physiology or synchrony metric can represent—and therefore what it can and cannot support.		
Isomorphism		A perfect one‐to‐one mapping: a physiological observation or pattern would uniquely identify a psychological state or process, and vice versa. This is rare, so most interpretations require strong theory, context, and convergent evidence to be defensible.		
*Functional Types of Psychophysiological Relationships*				
**Relationship type**	**Specificity**	**Generality**	**Plain language**	**Implication for physiological synchrony**
Outcome	Low	Low	Physiology changes in response to psychological activity, but the same physiological change can arise from many different psychological states. For instance, increased corrugator (“brow furrow”) activity can accompany anger, sadness, anxiety, or concentrated effort, and its meaning may shift across tasks and settings (Devine et al. 2023; Dimberg 1990; Jäncke 1996; Larsen et al. 2003).	Useful for documenting that physiological synchrony occurred, but insufficient on its own for strong psychological interpretation.
Marker	High	Low	A physiological response or pattern uniquely corresponds to a specific psychological process, but only in a restricted set of situations or populations. For instance, the BPS model posits that a specific pattern of cardiovascular changes can be used to mark psychological states of challenge and threat, but only in motivationally relevant contexts (Blascovich et al. 2003).	Supports stronger inference when synchrony is tied to a specific psychological process under clearly specified theoretical and contextual conditions.
Concomitant	Low	High	A physiological response or pattern is broadly associated with a set of psychological processes but not uniquely tied to any single one.	Likely describes many synchrony findings that index broad processes such as shared arousal, engagement, or regulatory demand without specifying precise psychological content.
Invariant	High	High	A physiological response or pattern uniquely and reliably corresponds to a psychological process across contexts.	Would provide the strongest possible basis for inference, but such relationships have not been clearly established and are unlikely to be common in psychophysiology.

*Note:* Adapted conceptually from Cacioppo and Tassinary's (1990) taxonomy of psychophysiological relationships. These definitions clarify how key inferential terms are used in the present framework. The functional relationship types are best treated as distinctions in the strength and scope of inference, rather than fixed properties of any physiological measure. Throughout, claims about psychological meaning are strongest when specificity, generality, and sensitivity jointly constrain interpretation and when those claims are supported by theory, context, and convergent evidence. Although the classical taxonomy describes markers and invariants in one‐to‐one terms, the present framework treats these categories as idealized functional distinctions; most empirical applications increase or decrease specificity rather than eliminating ambiguity altogether.

## 3 Psychophysiological Inference: Specificity and Generality as Inferential Constraints

Drawing psychological conclusions from synchronized physiological activity requires confronting longstanding challenges of psychophysiological inference, particularly those of *specificity* and *generality* (Cacioppo and Tassinary 1990). These challenges are amplified in physiological synchrony research because the focal observation is not a single person's physiological response, but coordination between physiological signals whose psychological relevance depends on the interpersonal process being studied. Contextual constraint is therefore an important inferential starting point: physiological synchrony observed during conflict, support, cooperation, threat, or affiliative interaction is not interpreted in a vacuum. At the same time, context alone rarely resolves the specificity problem. The same constrained context can plausibly elicit similar physiological activation for different psychological reasons, and the same psychological context may contain multiple interpersonal processes unfolding simultaneously. Physiological patterning therefore becomes especially important for addressing specificity by narrowing which psychological interpretations remain plausible.

We begin with a brief overview of the conceptual issues related to drawing psychophysiological inference and then focus substantial attention on specificity because it is the most underdeveloped problem in physiological synchrony research.^3^ We argue that increasing specificity involves two complementary strategies. First, physiological ambiguity can be reduced through measurement choices, such as moving from global physiological indices, like heart rate and interbeat interval (IBI) to branch‐specific measures of autonomic activity that more selectively reflect sympathetic versus parasympathetic influence. Second, psychological interpretation can be further constrained through theory‐specified psychophysiological profiles (i.e., convergent patterns across measures that distinguish candidate psychological processes). Such profiles may be formalized in different ways, including as modes of autonomic control (Berntson et al. 1991) or as other theory‐specified physiological profiles (Blascovich et al. 1999; Stellar et al. 2020). We then turn to the complementary challenge of generality, examining when and how interpretations of synchrony can extend across tasks, contexts, and populations. Next, we address sensitivity (i.e., detectability): how measurement and modeling choices determine whether the theoretically relevant features of physiological activity can be detected, including the extent to which the data and analytic approach can detect the form of physiological synchrony implied by theory. Finally, we map common physiological synchrony terms and model classes onto these inferential constraints.

## 4 Classical Principles of Psychophysiological Inference

Classical training in psychophysiology emphasizes that physiological data acquire psychological meaning only through careful hypothetico‐deductive reasoning that links physiological patterns to theoretical claims about psychological processes (Cacioppo and Tassinary 1990). A central challenge in this reasoning is the lack of isomorphism between psychological and physiological elements: a single physiological response can arise from multiple psychological states, and a single psychological state can recruit multiple physiological systems. For instance, an increase in heart rate can reflect threat, joy, cognitive effort, or thermoregulation (Berntson et al. 2016; Kreibig 2010; Levenson et al. 2016; Rowell 1974); conversely, “anxiety” can manifest across multiple physiological systems (e.g., cardiac, electrodermal, respiratory) rather than any single channel (Kreibig 2010). This many‐to‐many mapping necessitates disciplined inference rather than direct interpretation.

**FIGURE 1:**
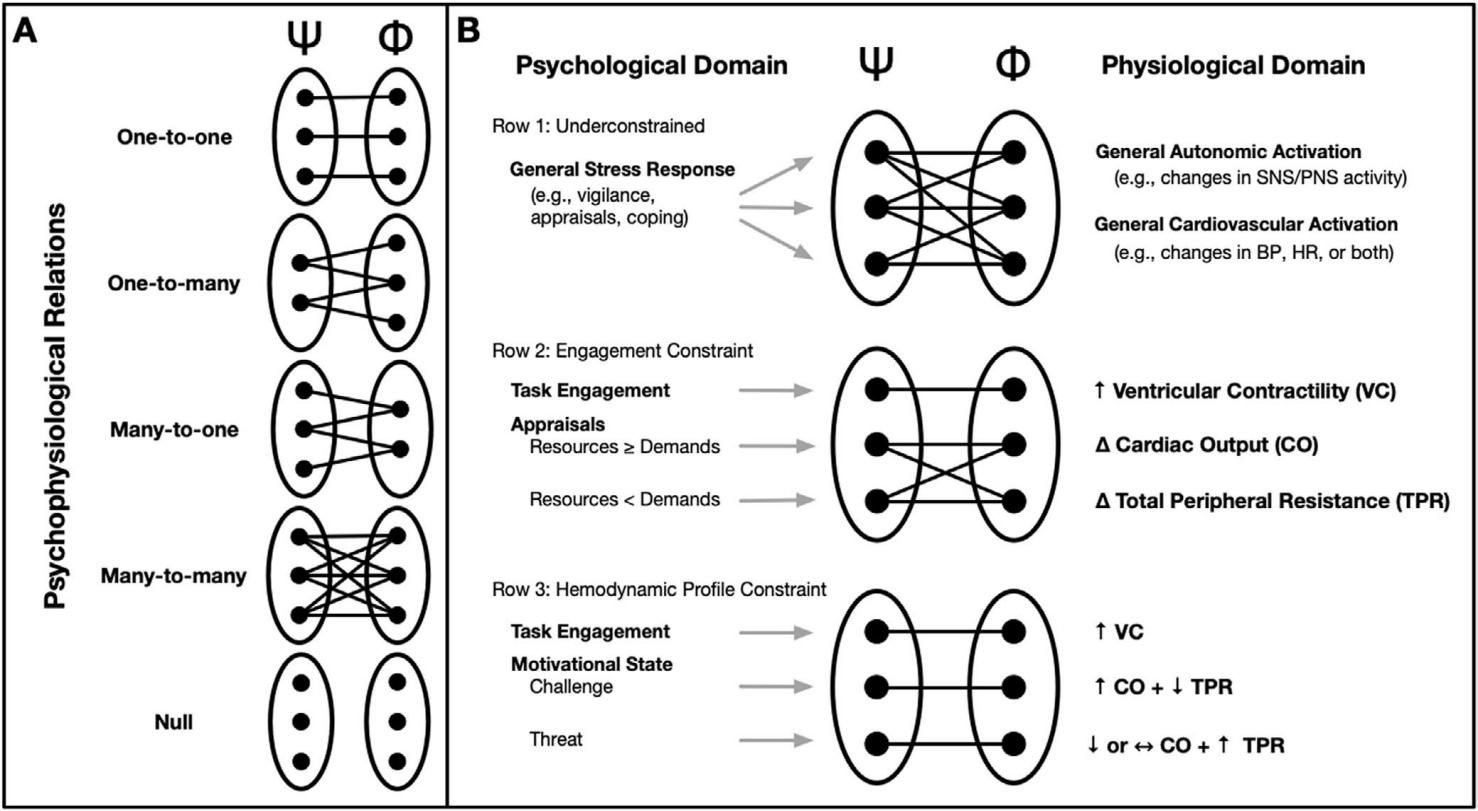
Conceptual schematic of psychophysiological mappings and theory‐guided constraint. Panel A is an original schematic, with all elements redrawn for the present manuscript, illustrating canonical forms of mapping between psychological (Ψ) and physiological (Φ) domains: one‐to‐one, one‐to‐many, many‐to‐one, many‐to‐many, and null mappings. Panel A is conceptually based on Cacioppo and Tassinary (1990), and its pedagogical presentation was informed by Page‐Gould (2019; https://doi.org/10.17605/OSF.IO/92FCQ). Panel B uses the biopsychosocial model (BPS) of challenge and threat as a worked example of theory‐guided constraint. In both panels, lines indicate possible mappings between psychological states and physiological patterns. In Panel B, each row depicts a progressively more constrained mapping; as theory‐guided constraints are added, the set of plausible mappings narrows, increasing specificity. Row 1 begins with a general stress response, in which psychological and physiological activation are broadly compatible but nondiagnostic. Row 2 adds a key eligibility constraint on the challenge–threat inference: motivated task engagement, indexed by increased ventricular contractility. This establishes that participants are actively engaged with the stressor and rules out interpretations that do not involve active engagement. Row 3 interprets the cardiac output (CO)–total peripheral resistance (TPR) hemodynamic pattern conditional on confirmed engagement: a challenge profile (↑CO, ↓TPR) supports the inference that perceived resources meet or exceed demands, whereas a threat profile (↓ or ↔CO, ↑TPR) supports the inference that perceived demands exceed resources. Arrows indicate direction of change relative to baseline or comparison condition; ↑ = increase, ↓ = decrease, ↔ = no meaningful change, and Δ = change.

To address this challenge, Cacioppo and Tassinary (1990) proposed that psychological and physiological states are more productively conceptualized as patterns of their constituent elements rather than isolated responses. Representing states as patterns allows scholars to specify the full range of hypothetical psychophysiological relationships between psychological and physiological elements, including one‐to‐one, one‐to‐many, many‐to‐one, many‐to‐many, and null relationships (Figure 1A). These hypothetical possibilities form the basis for evaluating the specificity of a psychophysiological relationship and, together with generality, provide the conceptual foundation for making valid psychophysiological inferences, including judgments about whether and when physiological synchrony carries psychological meaning.

## 5 Specificity: Mapping Physiology to Psychology

Specificity concerns the degree to which a physiological observation—whether a signal, state, event, or pattern—narrows the range of plausible psychological interpretations. Consistent with Cacioppo and Tassinary (1990), we use the term *element* to refer broadly to any psychological or physiological signal, state, event, or pattern that may enter into such a relationship. Five categories of hypothetical psychophysiological relationships can be distinguished:

- One‐to‐one. A psychological element corresponds to one and only one physiological element, and vice versa.
- One‐to‐many. A psychological element corresponds to a subset of physiological elements.
- Many‐to‐one. Several psychological elements correspond to the same physiological element.
- Many‐to‐many. Multiple psychological and physiological elements correspond in overlapping ways.
- Null. No systematic relationship exists.

Although these relationships are defined at the level of individual elements, higher‐order patterns of elements can also function as units of inference (see Figure 1B). A single physiological signal, state, or event may support broad psychological interpretation, but theoretically specified patterns can often narrow the range of plausible interpretations further. When a psychological pattern consistently corresponds to a physiological pattern, it can therefore support stronger, though still conditional, inference—even if individual elements do not exhibit one‐to‐one mappings. This distinction is particularly important for physiological synchrony, which is itself a patterned phenomenon.

### 5.1 Why Specificity Is Hard in Physiological Synchrony Research

Physiological synchrony magnifies the problem of specificity because it involves coordinated physiological activity across individuals rather than within a single person. In intrapersonal psychophysiology, ambiguity arises because a given physiological response can reflect multiple psychological states. In interpersonal contexts, this ambiguity is compounded: similar physiological changes observed in two people may arise from different psychological processes, serve different regulatory functions, or reflect distinct subjective experiences (Thorson et al. 2018). As a result, correspondence at the physiological level does not guarantee correspondence at the psychological level. For example, two strangers participating in the same ostensibly affiliative interaction may show coordinated sympathetic activation over time, yet that physiological alignment need not reflect a shared psychological state. For one partner, sympathetic activation may reflect excitement, novelty, or motivated approach toward a potentially rewarding interaction; for the other, it may reflect social anxiety, vigilance, or uncertainty during an unfamiliar interpersonal encounter. Thus, even within a single constrained context, similar patterns of physiological activation—and their apparent synchronization across partners—may arise from psychologically distinct processes.

Synchrony therefore introduces a second‐order inferential challenge. Researchers must determine not only what a given physiological change might mean within each individual, but also whether coordination across individuals reflects (a) a genuinely shared psychological state (i.e., convergent appraisal, shared affect, joint attention), (b) parallel but psychologically distinct responses, or (c) common‐input covariation driven by shared external constraints (e.g., task structure, pacing, posture, ambient conditions) rather than a shared internal state. This added layer of indeterminacy makes specificity particularly difficult to achieve in physiological synchrony research.

One way to reduce this indeterminacy is to shift the inferential target from single physiological signals to theory‐specified physiological profiles. Pattern‐based inference can increase specificity by requiring convergent changes across multiple components (and/or branches) that jointly distinguish candidate psychological processes—especially when common‐input accounts would not be expected to generate the full predicted pattern. In this sense, patterning functions as an inferential constraint: it narrows what physiological synchrony can plausibly mean before any one psychological label is applied.

To understand why these challenges arise—and how they might be addressed—it is necessary to examine the physiological systems in which coordinated physiological activity can emerge and be quantified as synchrony, with particular emphasis here on the autonomic nervous system (ANS; key terms are defined in Table 2). The structure and organization of the ANS introduce multiple sources of physiological ambiguity, such that the same observed signal (e.g., heart rate or IBI) may reflect different underlying regulatory or psychological states. When physiological activity is coordinated across people, this ambiguity is compounded, creating substantial challenges for psychological interpretation. Increasing specificity therefore requires constraints at multiple inferential levels, including measurement choices that reduce physiological ambiguity, modeling choices that determine which forms of coordination are estimable, physiological theories that organize regulatory patterns, and—where possible—psychological theories that link those patterns to meaning.

**TABLE 2:** Physiological systems and organizational principles: key terms and phrases in plain language.

Key terms/phrases	Plain language
Autonomic nervous system (ANS)	Neural pathways that regulate internal organ function, especially through sympathetic and parasympathetic outflows, supporting rapid adjustments in cardiovascular and other visceral processes.
Sympathetic nervous system (SNS)	A branch of the ANS that supports mobilization (e.g., increased cardiac output and vascular tone) during effort, stress, and active engagement.
Parasympathetic nervous system (PNS)	A branch of the ANS that supports restorative and regulatory processes and can reduce heart rate via cardiac parasympathetic (vagal) control.
Dual innervation	Many visceral organs are influenced by both sympathetic and parasympathetic inputs.
End‐point ambiguity	Because organs are dually innervated, the same observed endpoint (e.g., heart rate) can be produced by different configurations of SNS and PNS influence. The endpoint alone therefore does not identify the underlying autonomic configuration, limiting the psychological inferences it can support.
Branch‐specific indices	Measures intended to more selectively index branch‐specific influences on a physiological endpoint. For instance, pre‐ejection period (PEP) is commonly used as an index of cardiac sympathetic influence on ventricular contractility (measured from ECG and impedance cardiography). Respiratory sinus arrhythmia (RSA) is commonly used as an index of cardiac vagal (parasympathetic) influence on heart period variability and depends on respiratory parameters.
Doctrine of Autonomic Space	A framework describing how sympathetic and parasympathetic influences can be organized jointly into distinct modes of autonomic control, including reciprocal, coactive, coinhibited, and uncoupled configurations. These configurations and their implications for physiological synchrony are summarized in Table 3.

*Note:* These definitions are provided to clarify sources of end‐point ambiguity and to show how measurement choices can increase specificity by narrowing the range of plausible physiological and psychological interpretations. Even branch‐specific indices do not uniquely identify psychological states or processes; mind–body mappings are rarely isomorphic. Interpretation therefore remains dependent on theory, context, convergent evidence, and clearly specified conditions under which a given interpretation is warranted.

### 5.2 The Autonomic Basis of Physiological Synchrony

The autonomic nervous system (ANS) regulates the body's visceral organs, dynamically adjusting to both environmental demands and internal goals (Jänig 2006). In doing so, it supports psychological functions such as motivation (Blascovich et al. 2003; Kelsey 2012) and attention (Suess et al. 1994), producing physiological signals that—when measured—can reveal how social experience becomes embodied within and between people. Because many forms of physiological synchrony are observed in signals generated by autonomic regulation, interpreting physiological synchrony requires a basic understanding of ANS structure and organization. Without this foundation, physiological synchrony findings can be too easily mapped onto psychological labels that physiology cannot support.

#### 5.2.1 Dual Innervation and the Challenge of Ambiguity

Most visceral organs receive dual innervation from the sympathetic and parasympathetic branches of the ANS. Sympathetic nervous system (SNS) activation typically mobilizes the body for action—raising heart rate, contractility, and vascular resistance—whereas parasympathetic nervous system (PNS) activation promotes recovery and restoration, slowing heart rate and facilitating digestion (Jänig 2006). Crucially, these branches do not operate in simple opposition. A central implication of dual innervation is that the same physiological endpoint can be produced by different branch configurations. For instance, an increase in heart rate can arise from sympathetic activation, parasympathetic withdrawal, or coordinated change in both branches. The foundational framework for formalizing these configurations is the Doctrine of Autonomic Space (Berntson et al. 1991), which specifies how sympathetic and parasympathetic activity combine into distinct modes of autonomic control, including reciprocal, coactive, coinhibited, and uncoupled configurations.

This end‐point ambiguity creates a core specificity problem for physiological synchrony. Because heart‐rate synchrony is compatible with multiple underlying regulatory states, it cannot by itself specify the physiological source of synchrony, limiting what can be inferred psychologically. Researchers can reduce this ambiguity by using branch‐specific indices—such as pre‐ejection period (PEP) as an index of cardiac sympathetic influence (Kelsey 2012) and respiratory sinus arrhythmia (RSA) as an index of cardiac vagal, or parasympathetic, influence (Porges 2001). Physiological specificity can be sharpened further, where possible, when these branch‐specific indices are interpreted as part of broader autonomic configurations or other theory‐guided physiological profiles. Even then, branch‐specific changes and theory‐guided physiological profiles may remain compatible with multiple psychological interpretations, so stronger inference typically depends on a further layer of convergent evidence from psychological, behavioral, and contextual sources (e.g., self‐report, behavior, task demands, qualitative accounts). For example, if one hypothesizes that synchronized sympathetic activation reflects shared effort or threat, the interpretation is stronger when the interaction context is theoretically relevant and the same window shows parallel changes in theoretically linked measures, such as demand/resource appraisals, observed task engagement, self‐reported stress, or behavioral markers of vigilance. Such convergent evidence helps triangulate the inferred process rather than allowing the inference to rest on physiology alone.

A further constraint is that psychological interpretation is rarely carried by absolute autonomic level. Instead, it is typically tied to reactivity—change relative to each individual's baseline—because the same observed value of an autonomic index can be produced by multiple underlying configurations (baseline activity × person‐level characteristics × situational demands). For physiological synchrony, reactivity provides a first‐pass constraint on interpretation: coordination observed during reactivity is more plausibly linked to activation‐ or engagement‐related psychological processes, whereas coordination observed without substantial deviation from baseline provides weaker support for activation‐based interpretations and leaves open alternative explanations such as shared tonic regulation or common‐input covariation (e.g., posture, respiration/speaking patterns, pacing, ambient conditions). Activation‐based interpretations of physiological synchrony in the absence of marked reactivity therefore require stronger convergent evidence. At the same time, marked reactivity from baseline is not required for physiological synchrony to be socially meaningful or predictive. Synchrony requires within‐person variation over time to be estimable, but such coordination can predict social outcomes even when partners remain near baseline or when synchrony–outcome associations do not differ across activation levels (Danyluck and Page‐Gould 2019). Reactivity therefore qualifies interpretation rather than determining whether synchrony matters: when synchrony occurs without substantial deviation from baseline, interpretations that depend on strong activation (e.g., shared stress, threat, or effort) are less warranted, but other accounts—such as coordinated tonic regulation, affiliative attunement, low‐arousal social engagement, or shared task structure—may remain plausible if supported by theory and convergent evidence.

Baseline‐referenced change therefore reduces one source of indeterminacy by clarifying whether synchrony occurs during activation, deactivation, recovery, or relative stability rather than treating coordination around different autonomic levels as equivalent. However, another source of indeterminacy remains: even when synchrony is examined in branch‐specific indices and anchored in baseline‐referenced reactivity, autonomic branches are not isomorphic with psychological states. Thus, reactivity often becomes more informative when it is situated within the broader organization of autonomic control—or within another theory‐specified physiological profile. We take this issue up next by considering whether modes of autonomic control, and then theory‐specified profiles such as the biopsychosocial model of challenge and threat, can function as meaningful units of physiological synchrony.

### 5.3 Patterns of Autonomic Organization as an Inferential Constraint

Whereas the preceding sections established why branch‐specific measurement and baseline‐referenced reactivity can constrain interpretation, they also point to a further possibility: the relevant unit of coordination may not be a single signal or branch, but interpersonal correspondence in the broader organization of sympathetic and parasympathetic activity within autonomic space. Table 3 summarizes these autonomic configurations, such as reciprocal, coactive, coinhibited, and uncoupled activity, and clarifies why they matter for interpreting physiological synchrony.^4^ Rather than treating synchrony in a single endpoint as evidence of a specific psychological process, the table illustrates how different SNS‐PNS configurations can narrow, but not determine, psychological interpretation. Here we consider whether patterns of autonomic control can function as a more constrained unit of synchrony, clarifying when partners share broader regulatory organization and when apparent synchrony in one signal masks divergence in that organization.

**TABLE 3:** Autonomic configurations as constraints on interpreting physiological synchrony: key terms and phrases in plain language.

Autonomic pattern	Plain language description	Inferential value for synchrony research	Interpretive caution
SNS‐dominant reciprocal control	SNS activity increases while PNS activity decreases	Can narrow interpretations toward coordinated sympathetic mobilization with reduced parasympathetic restraint, especially in contexts involving effort, challenge, threat, vigilance, or active engagement.	This pattern should not be interpreted as a specific psychological state on its own; similar autonomic organization may accompany different forms of engagement depending on the task and interpersonal context.
PNS‐dominant reciprocal control	PNS activity increases while SNS activity decreases	Can narrow interpretations toward coordinated parasympathetic regulation with reduced sympathetic mobilization, especially in contexts involving recovery, calming, safety, or regulatory processes.	This pattern does not uniquely indicate calm or co‐regulation; it could also reflect disengagement, reduced task demand, or other context‐specific processes.
Coactive mode	SNS and PNS activity increase together	Highlights that synchrony may involve coordinated increases in both autonomic branches.	Because both branches increase together, endpoint changes such as heart rate or IBI may not reveal the underlying autonomic organization; interpretation remains dependent on branch‐specific measures, the social context, and convergent evidence.
Coinhibited mode	SNS and PNS activity decrease together	Highlights that synchrony may reflect joint withdrawal of autonomic influence.	Because both branches decrease together, endpoint changes such as heart rate or IBI may not reveal the underlying autonomic organization; interpretation remains dependent on branch‐specific measures, the social context, and convergent evidence.
Uncoupled SNS variation	Variation in SNS activity is not systematically related to changes in PNS activity	Indicates that synchrony may be branch‐specific, reflecting coordination in sympathetic influence without corresponding coordination in parasympathetic influence.	Lack of systematic coupling between branches does not mean the physiological pattern is meaningless; it means whole‐ANS interpretations should be avoided.
Uncoupled PNS variation	Variation in PNS activity is not systematically related to changes in SNS activity	Indicates that synchrony may be branch‐specific, reflecting coordination in parasympathetic influence without corresponding coordination in sympathetic influence.	Lack of systematic coupling between branches does not mean the physiological pattern is meaningless; it means whole‐ANS interpretations should be avoided.

*Note:* This table summarizes how different patterns of sympathetic and parasympathetic organization can increase specificity by narrowing the range of plausible interpretations of physiological synchrony. The configurations are described in terms of branch organization rather than endpoint direction. Coactive and coinhibited modes, like the other modes shown here, can be SNS‐dominant or PNS‐dominant depending on the relative influence of each branch on the endpoint being measured. Thus, a single endpoint such as heart rate or interbeat interval cannot, by itself, identify the underlying mode of autonomic control. For example, an increase in heart rate could reflect SNS‐dominant reciprocal control, SNS‐dominant coactivation, PNS‐dominant coinhibition, or uncoupled SNS variation. The purpose of this table is not to assign fixed psychological meanings to each autonomic configuration, but to show how branch‐specific measures, theory, context, and convergent evidence can narrow the range of plausible interpretations of physiological synchrony. Whether interpersonal alignment or divergence in these configurations improves psychological inference beyond single‐branch synchrony remains an open empirical question.

The possibility that partners may diverge in their broader autonomic organization matters because social partners can appear synchronized in one physiological signal while differing in the broader SNS‐PNS configurations that produced it. This problem is distinct from the psychological ambiguity discussed in preceding sections: the issue is not only that the same physiological response can reflect different psychological processes, but that the same apparent synchrony, when estimated from a single endpoint or branch, can arise from different SNS–PNS configurations across partners. For example, two partners may show synchronized variation in heart rate during a stressful interaction even if the branch configurations producing that variation differ. This ambiguity is not eliminated simply by moving from an endpoint such as heart rate to a branch‐specific measure. Two partners could show temporally coordinated sympathetic reactivity during a stressor while one simultaneously exhibits parasympathetic withdrawal, consistent with SNS‐dominant reciprocal control, and the other exhibits parasympathetic activation, consistent with coactivation. Measuring only SNS synchrony would identify their coordinated sympathetic mobilization while obscuring divergence in their broader autonomic organization.

There is some basis for considering whether such distinctions matter for psychological interpretation. At the individual level, nonreciprocal configurations (e.g., SNS–PNS coactivation or coinhibition) have been linked to poorer stress regulation and higher externalizing/aggressive outcomes in adversity‐relevant contexts, whereas reciprocal patterns during stress are often linked to better regulation (El‐Sheikh et al. 2009; Fanti et al. 2019; Suurland et al. 2018; Thomson et al. 2019). Related parent–infant research likewise suggests that multisystem physiological profiles can acquire different behavioral significance when interpreted jointly. Elevated maternal cortisol predicted greater negative intrusiveness only among mothers who showed smaller RSA reduction during a stressful reunion episode (Mills‐Koonce et al. 2009). Similarly, mothers of dysregulated infants showed elevated skin conductance without comparable parasympathetic withdrawal, whereas mothers of infants who recovered displayed parasympathetic withdrawal. Among mothers of dysregulated infants, this autonomic pattern was accompanied by less responsive behavior toward infants' disengagement cues (Ham and Tronick 2006). Although these studies do not validate mode‐level synchrony as a psychological marker, they illustrate the broader inferential point: the psychological or interpersonal interpretation of activity in one physiological system may depend on its configuration with activity in another. Applied to physiological synchrony, these findings raise the possibility that coordination in a single endpoint or branch may conceal physiologically meaningful alignment or divergence in partners' broader regulatory organization.

This possibility is inferentially consequential even if the precise psychological meaning of each autonomic mode remains unknown. If synchrony is interpreted as evidence that partners share a common psychological or regulatory process, that they are co‐regulating, or that one partner's response is transmitted to the other, evidence that their broader autonomic organization diverges would generally make that interpretation less defensible. At the same time, mode‐level alignment would not by itself establish that partners share a specific psychological state, nor would mode‐level divergence necessarily establish that they differ psychologically in the absence of convergent psychological or behavioral evidence. Individual‐level associations between autonomic modes and regulatory functioning therefore motivate, but do not validate, the use of autonomic modes in physiological synchrony research. Testing whether observed synchrony reflects alignment or divergence in modes of autonomic control shifts the inferential target from shared variation in a single signal to interpersonal correspondence in regulatory organization, providing a more precise physiological target for interpersonal research. Whether such correspondence discriminates among competing psychological accounts more effectively than single‐branch synchrony remains an open empirical question.

Yet this argument should not be read as implying that informative physiological synchrony requires simultaneous measurement of both autonomic branches in every study, or that single‐branch synchrony is uninformative. Uncoupled configurations are themselves regions of autonomic space and can be characterized by predominant change in one branch with relative stability in the other (e.g., parasympathetic withdrawal in the absence of sympathetic change). Thus, single‐branch synchrony can support constrained psychological inference when interpreted within appropriate contextual and theoretical boundary conditions and triangulated with non‐physiological evidence. Indeed, temporally structured, branch‐specific synchrony has been used to test interpersonal influence and transmission accounts under theoretically specified conditions, illustrating how single‐system synchrony can be informative when paired with convergent evidence (e.g., Waters et al. 2014; see Box 1).

BOX 1 **Constraining Psychological Inference From Single‐System Synchrony.**

Single‐system synchrony can support meaningful inference, but its inferential value depends on whether the psychological claim is appropriately scaled to the physiological evidence and whether the study design supplies additional constraints.

A strong example of constrained psychological inference from physiological synchrony comes from a study of physiological covariation between mothers and infants (Waters et al. 2014). The psychological construct—affect contagion—is broad enough for the physiological evidence: the authors are not claiming to identify a precise discrete emotion, but rather the transmission of an affective state defined in terms of valence and arousal. The experimental design also sharply constrains the originating state. Mothers were randomly assigned to positive social evaluation, negative social evaluation, or a non‐evaluative control task that preserved many physical and cognitive demands of the stressor while removing social evaluation. The manipulation was validated through maternal self‐reported affect and physiological reactivity, confirming that the evaluation conditions produced the intended affective and autonomic responses. Infant responses were then measured after reunion, when infants had not been exposed to the stressor directly. Infants of negatively evaluated mothers showed higher heart‐rate reactivity than infants in the control condition, and infants of socially evaluated mothers showed more avoidance toward strangers. Finally, mother–infant physiological covariation was strongest, and increased over time, in the negative‐evaluation condition. Thus, the physiological covariation finding is informative not because single‐branch synchrony uniquely identifies affect transmission, but because it is embedded within appropriate contextual and theoretical boundary conditions and triangulated with the experimental manipulation, manipulation checks, infant physiological reactivity, and infant behavioral evidence.

The same logic also clarifies the limits of single‐system inference. When single‐branch synchrony is paired with a strong experimental design and convergent evidence, it can support constrained psychological interpretation; when those contextual or behavioral constraints are less direct, the synchrony finding may be robust while its psychological meaning remains more ambiguous. This distinction is important because, in many single‐system studies, physiological synchrony may be demonstrated more clearly than its psychological interpretation is constrained. A useful illustration comes from research linking parent–child PNS synchrony to observed interaction behavior and discussing this coordination in relation to parent–child coregulation (Skoranski et al. 2017). Here, mother–child PNS synchrony, operationalized as time‐dependent RSA coordination, was stronger during greater maternal teaching and weaker during maternal disengagement. These findings are valuable because they show that single‐branch synchrony varies with meaningful features of interactional engagement. At the same time, they also raise the question of what additional evidence is needed before PNS synchrony can be interpreted as coregulation in the stronger regulatory sense. To establish physiological coregulation, the analysis would need to show that partners' physiological states were organized in a way that supported regulation—for example, that one partner's state or behavior predicted the other partner's movement toward a homeostatic set point, recovery following perturbation, reduced instability, or more effective behavioral engagement. The relevant behavioral evidence would also need to capture regulatory exchanges rather than engagement more generally. Maternal teaching and disengagement are plausible indicators of involvement, attention, and task engagement, but they do not directly identify processes such as soothing, comforting, repair, emotional support, or child regulation following maternal support. Stronger inference would require evidence that PNS synchrony was tied to these regulatory processes—for example, that child distress elicited maternal support, that maternal support predicted child behavioral or physiological recovery, or that PNS synchrony predicted independent indicators of child self‐regulation. This ambiguity is especially relevant because the regulatory interpretation of PNS withdrawal may depend on concurrent sympathetic activation (Skoranski et al. 2017). Thus, multisystem evidence may be needed to determine whether synchrony in one branch occurs within a broader autonomic configuration consistent with an interpretation of physiological coregulation.

The contrast illustrated in Box 1 clarifies why multisystem measurement is not a universal requirement, but a theory‐contingent tool for discriminating among otherwise similar physiological patterns. Concurrent SNS–PNS measurement may be especially valuable when theory makes it important to determine whether synchrony observed in one branch occurs within a broader uncoupled configuration, reflects alignment in a shared mode of autonomic control, or conceals divergence in partners' broader regulatory organization. In such cases, multisystem measurement can distinguish among physiological configurations that would otherwise appear similar in a single signal and can therefore support more discriminating psychological inference within constrained interpersonal contexts. If reliable, context‐specific mappings emerge, subsequent work may be able to use selected indices as pragmatic markers, provided that their inferential boundary conditions remain explicit.

#### 5.3.1 Analytic Considerations for Modeling Physiological Synchrony in Autonomic Modes

Modeling synchrony in autonomic modes requires more than adding multiple physiological streams to a conventional synchrony model. The relevant target is coordination in partners' evolving, branch‐specific patterns of autonomic organization: where each partner is located in autonomic space relative to baseline, how each partner moves through autonomic space over time, and whether those locations or movements align, diverge, or predict one another. Box 2 outlines one possible analytic approach.

BOX 2 **Modeling Physiological Synchrony as Shared Autonomic Modes and Transitions.**

A mode‐based analysis of physiological synchrony would involve three analytic decisions.

First, researchers would represent each partner's autonomic organization over time by retaining signed, branch‐specific SNS and PNS change within sufficiently reliable epochs. For early task epochs, the relevant reference point would ordinarily be baseline. For later epochs, researchers would need to preserve both the person's autonomic mode relative to baseline and the person's transition relative to the preceding epoch.

Second, researchers would define the synchrony target. Mode synchrony asks whether partners occupy similar autonomic modes at the same time. Transition synchrony asks whether partners show similar regulatory movements over time, even if they begin from, or remain in, different configurations. For example, partners may begin in different autonomic modes—one showing reciprocal sympathetic activation and the other coactivation—but both subsequently move toward the same reciprocal sympathetic mode. Lagged mode synchrony asks whether one partner's entry into, or movement toward, a particular mode predicts the other partner's subsequent mode or transition.

Third, researchers would select a model appropriate to the theoretical claim. Descriptive plots can illustrate partners' SNS and PNS trajectories, but they cannot formally estimate uncertainty, transition probabilities, dwell times, or partner‐linked state changes. Because physiological synchrony data are nested and temporally structured, multilevel hidden Markov models or related state‐switching approaches may offer useful tools when theory concerns movement among autonomic modes over time. Such models can, in principle, estimate latent autonomic modes, transitions among modes, and variability across individuals and dyads while accommodating hierarchical data structures (Mildiner Moraga and Aarts 2024; Pohle et al. 2021). However, applying these models to autonomic‐mode synchrony would require careful validation that inferred states correspond to theoretically meaningful SNS–PNS configurations and that partner‐linked transitions reflect interpretable autonomic coordination. Despite these demands, mode‐based analysis offers a clearer physiological target than covariation in a single physiological stream when theory concerns shared or diverging regulatory organization.

#### 5.3.2 Pattern‐Based Inference in the Biopsychosocial Model of Challenge and Threat

The preceding sections illustrate why achieving specificity in physiological synchrony research often requires progressively constraining inference through branch‐specific measurement, attention to baseline‐referenced reactivity, and theory‐specified autonomic profiles. To clarify how such constraints sharpen specificity in an established psychophysiological framework—and why stronger inference depends on clearly specified boundary conditions—it is useful to consider the biopsychosocial model of challenge and threat (BPS; Blascovich et al. 2003), in which psychological meaning is constrained by theory‐specified physiological patterns under explicitly specified conditions. Although the BPS model was developed to characterize intrapersonal stress responses, it provides a clear illustration of how theory‐driven convergence and contextual constraint can support strong inference (key terms are defined in Table 4). We use the BPS model here not as a model of physiological synchrony itself, but as an exemplar of the inferential logic relevant to physiological synchrony research. Specifically, it underscores the general point that specificity is often enhanced not through single signals alone, but through interpretable physiological configurations whose boundary conditions are specified a priori. Later, we return to this logic to consider whether coordinated challenge‐ or threat‐related profiles between partners might support more specific psychological interpretations in synchrony research.

**TABLE 4:** Key terms in the biopsychosocial model of challenge and threat.

Key terms	Plain language
BPS model of challenge and threat	A theory that distinguishes challenge vs. threat during motivated engagement based on appraisals (demands vs. resources) and theory‐specified cardiovascular response profiles across multiple indices (e.g., PEP, CO, TPR).
Motivated performance context	A context in which performance is goal‐relevant and engagement is motivated (often involving evaluation, uncertainty, or stakes), such that cardiovascular mobilization is expected (e.g., giving a speech).
Challenge (motivational state)	A motivational (psychological) state wherein the organism perceives themselves as having resources that meet or exceed the demands of a stressor.
Threat (motivational state)	A motivational (psychological) state wherein the organism perceives themselves as not having resources that meet the demands of a stressor.
Pre‐ejection period (PEP)	A commonly used index of cardiac sympathetic influence on ventricular contractility (measured from ECG and impedance cardiography). In the BPS framework, PEP shortening from baseline is used as evidence of task engagement in motivated performance contexts.
Cardiac output (CO)	The amount of blood pumped through the heart in liters/min. CO is a key determinant of oxygen and glucose available to brain and muscles; increases can support greater metabolic supply during active engagement.
Total peripheral resistance (TPR)	A measure of vascular resistance (higher values reflect greater constriction).
Challenge profile (pattern‐level summary)	Conditional on motivated task engagement, challenge is characterized by reduced PEP, increased CO, and reduced TPR.
Threat profile (pattern‐level summary)	Conditional on motivated task engagement, threat is characterized by reduced PEP, reduced or unchanged CO, and increased TPR.

*Note:* Challenge and threat inferences are intended for motivated performance contexts and are strongest when task engagement is evident, typically indexed by PEP shortening from baseline. Interpretation remains theory‐ and context‐dependent.

The BPS model distinguishes psychological states of challenge and threat not by overall arousal, but by coordinated patterns of cardiovascular reactivity that reflect underlying motivational appraisals (Blascovich et al. 2003; Figure 1B). In this framework, both challenge and threat involve sympathetic activation, but they diverge in vascular resistance and cardiac output, producing distinct profiles that reflect differences in the efficiency with which the cardiovascular system mobilizes metabolic resources. Crucially, neither state can be inferred from any single physiological measure; psychological interpretation depends on convergence across signals and on the presence of a motivationally relevant context—specifically, a motivated performance situation in which task engagement is evident (often elicited via social‐evaluative pressure), such as solving time‐pressured mental arithmetic problems or delivering a speech in front of an evaluator.

From the perspective of psychophysiological inference, the BPS model exemplifies a relationship with relatively high specificity and deliberately constrained generality. Specificity is increased through pattern‐based inference—linking coordinated changes in cardiac output, vascular resistance, and sympathetic activation to distinct psychological states—while generality is limited to contexts involving motivated performance or evaluative demand. Outside these contexts, similar physiological patterns may arise for different reasons, and strong inference may no longer be warranted.

This trade‐off illustrates a central principle of the present framework: strong psychological inference from physiology is possible, but only under explicitly constrained conditions. The BPS model does not eliminate ambiguity; rather, it manages ambiguity by specifying when particular physiological patterns are interpretable and when they are not. In doing so, it demonstrates how specificity and generality can be jointly addressed through theory‐specified physiological profiles and contextual constraint.

Although the BPS model was developed to characterize intrapersonal processes, its inferential logic has direct implications for physiological synchrony research. If strong psychological inference benefits from pattern convergence, contextual constraint, and explicit limits on generality within individuals, these considerations are likely even more stringent when interpreting coordinated physiological activity between people. From this perspective, many instances of physiological synchrony—particularly those observed in single measures or across heterogeneous contexts—may be best understood as supporting broader, less specific inferences unless additional constraints are imposed.

Modes of autonomic control and the BPS model are not intended as exhaustive accounts of physiologically meaningful patterning. Rather, they serve as illustrative examples of how theory can organize physiological signals into interpretable configurations. Other psychological theories—whether centered on motivation, affect, attention, or social dynamics—may define different patterns of coordinated physiological activity that afford inference under appropriate constraints. The present framework does not privilege any specific pattern; instead, it emphasizes the inferential logic required to justify psychological interpretation and to specify the boundary conditions under which that interpretation is warranted.

## 6 Generality: Situating Psychophysiological Relationships

As the preceding example illustrates, strong psychophysiological inference depends not only on how narrowly a physiological pattern constrains psychological interpretation, but also on the conditions under which that interpretation is defensible. Whereas specificity concerns the degree to which a physiological observation narrows the range of plausible psychological interpretations, generality concerns when and where those interpretations are warranted—an especially important consideration for physiological synchrony, which has been observed across diverse tasks, relationships, and contexts. A psychophysiological relationship may be relatively context‐free, emerging reliably across situations and individuals (Hawkley et al. 2003), or context‐dependent, appearing only under specific conditions (Danyluck and Page‐Gould 2018). Without attending to generality, even relatively specific relationships risk being misinterpreted or overgeneralized.

## 7 Combining Specificity and Generality: A Taxonomy of Psychophysiological Relationships

Together, specificity and generality define the inferential status of a psychophysiological relationship. Cacioppo and Tassinary's (1990) taxonomy distinguishes psychophysiological outcomes, markers, concomitants, and invariants according to whether physiological observations provide relatively narrow or broad psychological constraint and whether those relationships hold across many or only under restricted contexts (Table 1). Applied to physiological synchrony, this taxonomy helps calibrate the strength and scope of psychological inference. Some synchrony findings may function primarily as outcomes, documenting that physiological coordination occurred without strongly constraining its psychological meaning. Others may function as concomitants, indexing broad processes such as shared arousal, engagement, or regulatory demand across contexts. More constrained cases may function as markers when synchrony is tied to a specific psychological process within clearly specified theoretical and contextual boundary conditions. True invariants, in which a physiological pattern uniquely and reliably identifies a psychological process across contexts, remain largely hypothetical in psychophysiology.

This taxonomy underscores a central claim of the present framework: physiological synchrony is not itself a psychological construct, but an empirical pattern whose meaning depends on inferential constraints grounded in specificity, generality, and theory. The same logic applies when the construct of interest is not a discrete internal state or downstream outcome, but an unfolding biobehavioral process (Feldman 2012; Gordon et al. 2024). In such accounts, physiological activity and socially meaningful behavior may be reciprocally organized over time, such that similar physiological synchrony supports different psychological interpretations depending on whether it occurs during mutual engagement, escalating conflict, withdrawal, repair, or other unfolding interpersonal dynamics. This temporal framing introduces a third inferential consideration: whether the physiological measures, behavioral measures, and analytic models are sensitive to the temporal dynamics of the psychological process under study.

## 8 Sensitivity as a Temporal Constraint on Interpersonal Inference

Having established how specificity and generality constrain what physiological synchrony can mean and when those meanings hold, we now turn to a complementary requirement for psychological inference: whether the temporal dynamics of physiological coordination can be detected with sufficient sensitivity to support those inferences, including behavioral, contextual, or interpersonal features where theoretically relevant.

In classical psychophysiological theory, sensitivity refers to how well a physiological measure detects changes in a psychological process (Cacioppo and Tassinary 1990). Sensitivity is therefore primarily a property of measurement and design: it concerns whether a physiological signal changes when a psychological process changes, and whether that change is detectable given the signal's temporal resolution, signal‐to‐noise characteristics, and alignment with the time course of the process (e.g., rapid shifts in attention versus slower changes in stress appraisal). Importantly, sensitivity is not limited to response magnitude; it also concerns whether a measure can track the time course of a psychological process, including features such as onset, latency, duration, and sequencing—that is, whether theorized steps unfold in the expected order.

Applied to interpersonal physiology, sensitivity concerns whether a given measurement and analytic approach can detect coordinated dynamics across individuals, given the timing and structure of the underlying interpersonal psychological process. Decisions about sampling rate, preprocessing (e.g., smoothing/downsampling), observation window, and whether synchrony is tested as simultaneous or lagged determine which forms of coordination are detectable—and which candidate psychological processes are therefore eligible for inference (Palumbo et al. 2017). Relatedly, operational choices, such as whether synchrony preserves sign, can obscure theoretically meaningful distinctions in coordination. If key features of the data (e.g., temporal grain) or the operationalization (e.g., whether synchrony preserves sign) cannot resolve the dynamics implied by a theory (e.g., rapid reciprocal adjustment versus slower covariation), the absence—or presence—of “synchrony” is not interpretable in psychological terms. This requirement is especially consequential when theory specifies a dynamic relation between physiological synchrony and behavior (Feldman 2012; Gordon et al. 2024). In such accounts, behavior is not merely a downstream outcome or an external source of convergent evidence; it may mark the interpersonal episode in which physiological synchrony is expected to emerge, provide a concurrently coordinated signal, precede or follow changes in physiological synchrony, or participate in a reciprocal feedback process. For example, physiological synchrony observed during behavioral engagement, conflict escalation, withdrawal, or repair may support different psychological interpretations even when its formal features—such as magnitude, sign, lag, or directionality—appear similar. Testing such accounts therefore requires temporal alignment between physiological and behavioral measures at a resolution capable of distinguishing the proposed sequence or coupling. A synchrony estimate aggregated across an interaction may detect overall physiological coordination while remaining insensitive to whether coordination was stable across the episode, concentrated around specific events, contingent on one partner's behavior, or organized as a sequence of physiological and behavioral changes.

### 8.1 Temporal Aggregation and Windowing

One major source of this sensitivity problem is temporal aggregation. Decisions about the temporal windows used to process, score, and aggregate physiological data shape the temporal grain at which synchrony can be detected and interpreted. Longer windows may yield more reliable estimates of some derived physiological measures, whereas shorter windows may better preserve rapidly unfolding interpersonal and autonomic dynamics but reduce reliability or specificity. Windowing decisions should therefore be guided by the temporal structure of the psychological or interpersonal process, the temporal dynamics of the physiological system and index being analyzed, and the reliability of the derived physiological measure.

This trade‐off is especially important for the present framework because the measures that can be modeled most easily at fine temporal resolution are not always the measures that most strongly constrain psychophysiological inference. Heart rate, IBI, and electrodermal activity (EDA) can often be modeled dynamically and may be well suited to questions about rapid physiological synchrony, arousal, or engagement. However, as already noted, heart rate and IBI are endpoints that can reflect multiple combinations of sympathetic and parasympathetic influence (Berntson et al. 1991), whereas EDA indexes sympathetic activity in a system from which a corresponding parasympathetic index is not available (Dawson et al. 2017). As a result, these measures are less suited to questions about coordinated SNS–PNS organization within a single dually innervated physiological system. By contrast, PEP and RSA are more informative when the theoretical question concerns branch‐specific sympathetic and parasympathetic influences within that system, but these measures have traditionally required larger scoring windows to achieve reliable estimation (Blascovich et al. 2011). Thus, synchrony research faces a recurring tension between temporal resolution and physiological specificity: the measures that are easiest to model dynamically may be less informative about branch‐specific autonomic organization, whereas measures that better constrain autonomic inference may require more aggregation.

Recent approaches such as moving ensemble estimation for PEP (Cieslak et al. 2018) and time‐varying RSA estimation (Gates et al. 2015) may help manage this tension because they preserve more temporal information while addressing some reliability problems that arise when estimating branch‐specific cardiac autonomic indices from very short segments. However, these approaches do not eliminate the sensitivity problem; they relocate it into decisions about the creation and interpretation of time‐varying estimates. Researchers must decide how long each moving window should be, how much adjacent windows should overlap, whether observations within a window should be weighted equally or weighted toward the center of the window, and how far the window should move before the next estimate is calculated. These choices determine not only the apparent temporal resolution of the resulting time series, but also the period of physiological activity each estimate represents and whether that estimate is reliable and valid for the physiological process it is intended to capture.

For instance, second‐by‐second RSA estimation is a meaningful advance for studying parasympathetic dynamics because it allows parasympathetic cardiac control to be modeled dynamically rather than only as an aggregate value across long epochs. However, this advance does not make RSA interpretation independent of the physiology from which RSA is derived. Because RSA remains constrained by respiratory gating, its dynamic interpretation depends on temporal parameters that correspond to the respiratory‐frequency range (Fisher et al. 2016). Indeed, finer‐grained temporal output is most informative when it is anchored to physiologically meaningful timing. The key point is that higher‐density estimates do not automatically yield more precise inferences. Even when measures can be estimated dynamically, researchers must still make methodological choices that map onto the conceptual and physiological signal of interest. The relevant question is therefore not only how often estimates are produced, but whether each estimate adequately represents the physiological event, interval, or dynamic process being linked to psychological or interpersonal theory.

A related temporal constraint arises in the estimation of synchrony itself. Up to this point, the discussion has focused primarily on how windowing affects the estimation of physiological indices, such as RSA or PEP. However, synchrony models face an additional sensitivity problem: the coupled component of two physiological signals can be small relative to the total variation within each individual (Altmann et al. 2022; Palumbo et al. 2017; Schoenherr et al. 2019). As a result, estimating synchrony typically requires analytic choices that separate shared temporal structure from larger uncoupled or asynchronous fluctuations (Behrens et al. 2020; Boker et al. 2002). Put simply, synchrony estimation is partly a signal‐separation problem: the researcher is trying to retain shared components of at least two signals while attenuating the much larger unshared variance. In time‐domain models, apparent interpersonal synchrony can arise because physiological signals are themselves autocorrelated, slowly varying, nonstationary, or jointly responsive to external events. Stronger inference therefore requires adequately representing each person’s own temporal structure and determining whether another person’s physiology explains additional temporal variation beyond it. A fundamental constraint from time–frequency analysis is that one cannot simultaneously localize a process precisely in time and isolate it narrowly in frequency (Cohen 2001; Gabor 1946). Consequently, synchrony estimated over short windows or rapidly changing episodes will necessarily be spectrally broad and more vulnerable to leakage from asynchronous components—whereas narrow‐band inferences require longer windows that sacrifice temporal specificity.^5^ This unavoidable trade‐off places an upper bound on how “selective” synchrony extraction can be and helps explain why window length, smoothing/downsampling, and model class materially change what synchrony is detectable and interpretable (Altmann et al. 2022; Schoenherr et al. 2019). When asynchronous variance dwarfs the coupled component, even small residual contamination—which is unavoidable given time–frequency limits—can substantially affect estimated synchrony (Altmann et al. 2022; Odenweller and Donner 2020). Across both time‐ and frequency‐domain approaches, then, seemingly minor preprocessing and modeling decisions can have outsized consequences for what physiological synchrony is detectable and, in turn, which psychological inferences are warranted.

### 8.2 Synchrony Is Not Arousal

**FIGURE 2:**
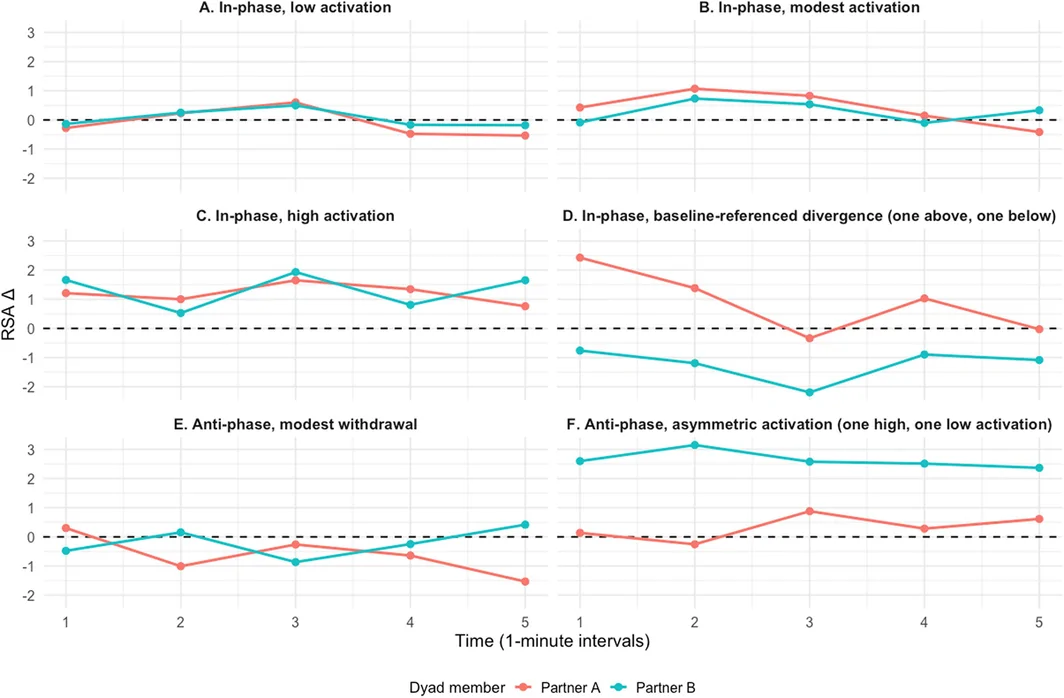
Synchronized fluctuations in parasympathetic nervous system (PNS) activity around baseline in romantic dyads (unpublished data). Each panel shows within‐dyad changes in parasympathetic activity over a 5‐min interaction (one‐minute segments). The y‐axis is RSA Δ (i.e., PNS activity relative to each individual's baseline). The dashed line indicates zero change from baseline. Colored lines represent the two dyad members. Panels are selected as exemplars to illustrate that physiological synchrony is distinct from activation context (i.e., baseline‐referenced activation), and that the interpretability of synchrony depends on direction and magnitude of deviation from baseline. Panel A shows in‐phase synchrony under low activation (both partners remain near baseline); Panel B shows in‐phase synchrony under modest activation; Panel C shows in‐phase synchrony under high activation; Panel D shows in‐phase synchrony with baseline‐referenced divergence (one partner mostly above baseline, the other consistently below); Panel E shows anti‐phase synchrony around modest withdrawal; and Panel F shows anti‐phase synchrony with asymmetric activation (one partner highly activated, the other around baseline). These panels are illustrative rather than exhaustive; other synchrony forms (e.g., lagged) are also possible and may support different psychological interpretations.

Sensitivity considerations also highlight a basic but consequential distinction: synchrony is not arousal. Synchrony describes whether partners' physiological signals change together over time; it does not, by itself, index the level of physiological activation around which that synchrony occurs—a distinction previewed earlier in relation to baseline‐referenced reactivity. This distinction matters for specificity and generality because the psychological interpretation of physiological synchrony may depend on the activation context in which it occurs, such as whether partners are above baseline, near baseline, below baseline, or recovering from activation. As a result, partners may exhibit strong synchrony while remaining close to baseline (Figure 2A) and, conversely, may be highly activated while showing little synchrony. Because many physiological indices can carry different psychological implications depending on baseline‐referenced reactivity or recovery, commonly modeled features of synchrony, such as magnitude, sign, lag, and directionality, may be insufficient for distinguishing among these interpretations. The issue is therefore not only whether synchrony occurs, but whether the study design and analytic plan preserve the activation, deactivation, or recovery dynamics needed to interpret that synchrony psychologically. In practice, however, many physiological synchrony analyses do not model activation context in conjunction with synchrony magnitude, which can obscure which psychological interpretations of physiological synchrony are warranted.

When activation context is modeled alongside physiological context—that is, the physiological system or configuration in which synchrony occurs—the interpretive stakes become clear. In a study of stranger dyads, physiological synchrony occurred across conditions in both autonomic branches, but prediction of affiliation depended on both the activation context and the physiological context of synchrony, not the mere existence of synchrony (Danyluck and Page‐Gould 2019). Specifically, sympathetic synchrony predicted perceived similarity independent of sympathetic reactivity, whereas parasympathetic synchrony's association with friendship interest depended on parasympathetic reactivity and the social context of the interaction.

This interpretive problem becomes even sharper when partners' changes are coordinated but differ in direction relative to baseline. Two partners can covary tightly over time while occupying different baseline‐referenced activation levels (e.g., one consistently above baseline, the other below; Figure 2, Panels D and F). Such a configuration can yield statistically strong synchrony yet plausibly reflect qualitatively different—or even opposing—psychological states rather than a shared affective or regulatory state. For instance, baseline‐referenced increases in RSA are often interpreted as greater parasympathetic engagement (e.g., calmer, socially engaged, or less effortful attention; see Appelhans and Luecken 2006; Porges 2001), whereas decreases are often interpreted as parasympathetic withdrawal (e.g., stress, vigilance, or focused effort; see Beauchaine 2015; Cacioppo et al. 1978; Suess et al. 1994; Thayer and Lane 2000). A second possibility is that partners may experience a broadly similar psychological state yet differ in baseline‐referenced physiological activation because stable individual differences can shape (and sometimes constrain) the magnitude and organization of their reactivity, including patterns consistent with adversity‐linked calibration of autonomic control (El‐Sheikh et al. 2009; Fanti et al. 2019; McLaughlin et al. 2014; O'Riordan et al. 2023; Suurland et al. 2018; Thomson et al. 2019). Although baseline is often a useful reference point for interpreting physiological activation, in some designs the relevant activation context may be baseline activity itself, recovery from a stressor, or movement toward or away from a previously established physiological state, such as a change in autonomic mode.

Accordingly, the psychological interpretation of physiological synchrony is strengthened when researchers consider, where possible, (a) physiological context or configuration, such as the branch‐specific index or theory‐specified physiological profile in which synchrony is modeled; (b) activation context, defined with reference to a theoretically meaningful physiological state or comparison point for each partner; and (c) direction of deviation from that reference point, such as increase versus decrease relative to the comparison point. Once the relevant physiological context and comparison point are specified, researchers can model activation context in several ways. In baseline‐referenced designs, this may involve including baseline levels as covariates, modeling baseline‐centered time series or other reactivity/change scores, testing whether baseline‐referenced activation moderates the association between physiological synchrony and psychological, behavioral, or relational outcomes (e.g., Danyluck and Page‐Gould 2019), or stratifying synchrony estimates by activation context when theory predicts different meanings for coordinated increases, decreases, or minimal deviations from baseline. In this sense, a stronger interpretation of physiological synchrony may depend not only on its formal features, but also on the physiological context in which synchrony is estimated and partners' activation contexts relative to the relevant reference point. Analogous approaches can be used when the relevant reference point is not baseline, such as change from a prior stressor, a preceding interaction epoch, or a previously identified autonomic configuration.

Even when activation context is modeled, stronger inference typically requires convergent evidence beyond physiological data, such as self‐report, behavioral, contextual, and qualitative evidence. These forms of evidence help constrain the psychological interpretation of physiological synchrony, but their inferential value also depends on temporal alignment: they must be sampled, coded, or aggregated at a resolution that preserves their relation to the physiological dynamics being interpreted. For example, when the theoretical claim concerns dynamic biobehavioral coordination, researchers need study designs and analytic approaches capable of preserving the unfolding psychological, behavioral, and physiological processes through which synchrony arises, changes, or acquires meaning (Feldman 2012). These temporal relations are difficult to evaluate when physiological synchrony is treated as a single summary quantity or when psychological, behavioral, and physiological data are aggregated at a temporal resolution too coarse to preserve their ordering. In practice, measurement, preprocessing, aggregation, and modeling choices determine which formal features of synchrony are detectable, such as magnitude, sign, lag, and directionality, and whether those features can be interpreted in relation to activation context. These choices therefore carry inferential commitments about what form of physiological synchrony is being tested and what psychological processes remain eligible for interpretation. To make these commitments explicit, we next map common physiological synchrony terms onto the inferential constraints they primarily implicate before turning to model class as an additional inferential constraint on which forms of physiological synchrony can be estimated, represented, and interpreted.

## 9 Mapping Physiological Synchrony Terms Onto Inferential Constraints

Physiological synchrony research routinely describes synchrony using terms such as magnitude, sign, directionality, lag, and observation window (Palumbo et al. 2017; key terms are defined in Table 5). What has been largely absent, however, is an explicit mapping of these descriptive features onto the inferential constraints long emphasized in classical psychophysiology. Table 6 provides this translation by summarizing how formal features of physiological synchrony constrain psychological inference through specificity, generality, and sensitivity.

**TABLE 5:** Features of physiological synchrony/interpretive qualifiers: key terms and phrases in plain language.

Theme	Key terms/phrases	Plain language
Synchrony features	Magnitude	How strongly partners' physiological signals are coordinated over time (how “in sync” they are), regardless of overall activation level.
	Sign	Whether partners' signals move together (in‐phase) or in opposite directions (anti‐phase). Sometimes described as concordant and discordant synchrony.
	Lag	The time delay(s) over which synchrony is assessed (e.g., contemporaneous vs. delayed alignment at a specified lag).
	Directionality	Whether coordination is symmetric or suggests one partner tends to lead and the other follow, though not proof of causality. It may be unidirectional, bidirectional, or multidirectional.
Temporal and sampling features	Observation window	The time span used to estimate synchrony (e.g., 30 s, 2 min). Shorter windows capture faster dynamics but are noisier; longer windows are more stable but can blur short‐lived effects.
	Temporal grain	Effective time resolution after preprocessing/windowing (e.g., second‐by‐second vs. 30‐s averages).
	Sampling rate	How frequently the signal is recorded (sets the fastest changes you can detect).
Interpretive qualifiers	Physiological context	Which physiological system or branch the synchrony is measured in (e.g., SNS vs. PNS indices; endocrine or neural measures).
	Activation context	The level or state of physiological activation in which synchrony occurs, such as baseline activity, activation or withdrawal relative to baseline, recovery from a prior stressor, or change relative to a preceding epoch.
	Baseline‐referenced reactivity	How much partners deviate from baseline (magnitude of change).
	Direction relative to baseline	Whether each partner is above vs. below baseline (direction of change).
	Common‐input covariation	Apparent synchrony driven by shared external factors (e.g., pacing, speaking/respiration, posture, environment) rather than interpersonal linkage.

*Note:* The themes in this table organize common terms by their descriptive function in synchrony research; they are not themselves mutually exclusive inferential constraints. “Interpretive qualifiers” refers to contextual or physiological information that changes what a synchrony estimate can mean, constraining inference through specificity, generality, and sensitivity depending on how it enters the design, measurement, and analysis. Psychological interpretation ultimately depends on theory, context, and convergent evidence.

**TABLE 6:** Mapping key physiological synchrony features onto psychophysiological inferential constraints.

Physiological synchrony feature (common term)	Operational definition	Inferential constraint(s) implicated (primary; secondary)	How it constrains inference	Threats to inference (interpretive + methodological)
Magnitude	Strength of covariation/synchrony between partners' signals over time as estimated by a specified model/estimator (e.g., correlation, synchrony parameter).	Primarily descriptive; Specificity (secondary, only when a theory predicts magnitude differences and alternatives are addressed); Sensitivity (secondary, via detectability/estimation precision).	Describes the strength of synchrony estimated by a specified model class/estimator, given the data and preprocessing pipeline. Magnitude can vary with estimator choice and preprocessing/windowing because these choices alter the relative contribution of shared (coupled) versus uncoupled variance (signal‐to‐noise). **Specificity:** Can contribute only when theory predicts magnitude differences across conditions and plausible alternatives, especially common‐input structure, are measured/controlled. **Sensitivity:** Larger magnitudes are generally estimated more reliably and precisely at a given sampling rate and observation window; small magnitudes may be unstable across reasonable analytic specifications.	**Interpretive:** Treating higher synchrony magnitude as inherently “more arousal,” “more connection,” or “more meaningful.” Magnitude can reflect many different psychological processes (or none), and can be driven by shared task/environmental structure as well as interpersonal processes. Interpreting magnitude as interpersonal “connection” is especially unwarranted without convergent evidence and without addressing common‐input alternatives. **Methodological:** Ignoring signal‐to‐noise constraints. Synchrony magnitude often reflects a small shared component embedded within much larger idiosyncratic variance (within‐person fluctuations, measurement noise, movement artifacts). When shared variance is small, estimated magnitude becomes highly contingent on preprocessing choices that alter the relative mix of shared versus unshared variance. Filtering can inflate or attenuate synchrony depending on which frequency components are retained; detrending can remove (or reveal) shared slow drift that would otherwise drive correlations; and window length can average away transient coordination or allow slow common fluctuation to dominate (and can obscure phase‐specific changes in synchrony). As a result, modest analytic differences can produce meaningfully different synchrony magnitudes, threatening inference unless synchrony estimates are shown to be robust across reasonable preprocessing/windowing choices and common‐input drivers are measured/controlled.
Sign (in‐phase vs. anti‐phase)	Positive versus negative synchrony (signals move together versus in opposition), as estimated by a specified model/estimator.	Specificity (primary); Generality (secondary).	Indicates whether coordination is convergent (in‐phase) or opposing/complementary (anti‐phase). **Specificity:** When theory specifies whether coordination should be convergent versus compensatory, sign narrows the interpretive space by making some candidate mechanisms more or less plausible (e.g., shared versus complementary dynamics). When theory is underdeveloped, sign can still be informative as a descriptive regularity that motivates competing hypotheses, but it does not by itself identify a unique psychological process. **Generality:** The same sign can plausibly carry different meanings across tasks, relationships, and role structures.	**Interpretive:** Treating sign as inherently “good” (in‐phase) or “bad” (anti‐phase), or as uniquely diagnostic of a single process without specifying task demands and mechanism. **Methodological:** Using sign‐insensitive metrics that collapse in‐phase and anti‐phase coordination; when using phase‐insensitive group‐level indices, interpret them as overall coordination strength rather than evidence about convergent versus complementary dynamics. Treating sign as time‐invariant when coordination may vary within an interaction; where theory implies within‐episode shifts, use time‐localized estimates and report dependence on reasonable windowing/preprocessing choices.
Directionality (Unidirectional: A → B, B → A; bidirectional; multidirectional)	Asymmetric versus symmetric synchrony (who predicts whom), including unidirectional (A → B or B → A), bidirectional (A↔B), and in group settings, multidirectional synchrony patterns.	Specificity (primary); Sensitivity (secondary); Generality (secondary, when contingent on roles/structure).	Directional asymmetries constrain which interpersonal processes remain plausible (e.g., influence/transmission, differential attunement, leadership or status‐linked coordination) versus mutual coordination. **Specificity:** Asymmetry (A → B ≠ B → A) is more consistent with accounts that posit directional influence and makes purely symmetric coordination accounts less plausible, given the theorized mechanism. **Sensitivity:** Directionality is interpretable only when the data and model can resolve temporal ordering (sampling rate, lag structure, window length) and when the estimator can represent asymmetry. **Generality:** Directionality may vary with relationship structure (e.g., status/expertise), context (e.g., conflict versus collaboration), or group topology.	**Interpretive:** Treating directionality as purely methodological rather than psychologically meaningful patterning; equating statistical directionality with causal influence; treating asymmetry as interpersonal influence without addressing common‐input or third‐variable structure. **Methodological:** Estimating directionality with insufficient temporal resolution or inappropriate lag/window choices; using models that impose symmetry or do not permit directional parameters; comparing directionality estimates across model classes that operationalize “prediction” differently.
Lag structure (simultaneous covariation vs. lagged)	Whether synchrony is estimated as simultaneous covariation (lag 0) versus lagged linkage (lag k), and the timescale of the lagged relation.	Sensitivity (primary); Specificity (secondary); Generality (secondary, context‐dependent).	Describes whether coordination is simultaneous or time‐lagged, and at what delay. **Sensitivity:** Whether sampling rate, preprocessing, and window length can resolve the theorized timescale of coordination. **Specificity:** Lagged linkage can make influence or transmission accounts more plausible, whereas purely simultaneous covariation is more consistent with shared fluctuation or common‐input accounts unless additional constraints are satisfied. **Generality:** Lag patterns may vary across tasks, relationships, and timescales, so interpretations should be bounded accordingly.	**Interpretive:** Treating any lag as evidence of influence/transmission without triangulating with design/context and without addressing common‐input structure. **Methodological:** Selecting lag(s) post hoc or across many candidate lags without principled constraints or correction for multiplicity; interpreting lagged effects that the effective temporal resolution cannot support (e.g., lags shorter than the sampling interval or shorter than the effective resolution after smoothing/downsampling), or lags that are unstable across reasonable window specifications.
Timescale	The temporal scale at which the interpersonal or psychological process is theorized to unfold, such as moment‐to‐moment adjustment, sustained coordination across an interaction, recovery across hours, or covariation across days.	Sensitivity (primary); Generality (primary); Specificity (secondary).	Determines which psychological processes are eligible for inference and the timescale at which an interpretation is warranted. A synchrony effect observed over seconds may support different interpretations than a similar effect observed over minutes, hours, or days. **Sensitivity:** The measures, sampling rate, and model must be capable of resolving dynamics at the timescale specified by theory. **Generality:** Synchrony estimated at one timescale is not automatically comparable to synchrony estimated at another; interpretations warranted at a micro‐timescale may not generalize to macro‐timescale coordination, and vice versa. **Specificity:** Timescale can narrow the range of plausible interpretations by distinguishing processes that unfold at different temporal scales, such as rapid reciprocal adjustment, sustained co‐fluctuation, recovery, or day‐level carryover.	**Interpretive:** Assuming the same psychological process operates across mismatched timescales; treating synchrony as a unitary construct across seconds, minutes, hours, days, or repeated episodes. **Methodological:** Using measures, sampling schedules, or models whose temporal resolution does not match the timescale of the theorized process.
Observation window/temporal aggregation	Time span or aggregation procedure used to estimate synchrony within the chosen timescale, such as fixed bins, moving windows, overlapping windows, or averaged epochs.	Sensitivity (primary); Specificity (secondary).	Determines which dynamics are detectable within the chosen timescale and which features of coordination remain visible. **Sensitivity:** Short windows may preserve transient coordination but can reduce reliability; long windows may yield more stable estimates but can miss brief dynamics, wash out phase‐specific synchrony, or allow slow shared drift to dominate. **Specificity:** Windowing and aggregation choices can affect whether theoretically relevant features, such as sign, lag, directionality, or event‐linked changes, remain available to narrow psychological interpretation.	**Interpretive:** Assuming a synchrony estimate reflects the theorized process without considering whether the window captures the relevant event, sequence, or temporal structure. **Methodological:** Choosing window length or aggregation strategy based on convenience or maximizing effects; failing to justify windowing decisions in relation to theory or test robustness across plausible alternatives.
Sampling rate/temporal resolution	Frequency with which the physiological signal is recorded and the temporal detail that remains after preprocessing steps such as downsampling or smoothing.	Sensitivity (primary).	Sets the upper limit on how precisely timing, lag, and directionality can be estimated. **Sensitivity:** Timing claims are warranted only at timescales the data can resolve; downsampling and smoothing can reduce effective temporal resolution and change which coordination structures remain detectable even when the original sampling rate is high.	**Interpretive:** Making timing claims (lags, rapid reciprocity) that the data cannot support. **Methodological:** Over‐interpreting temporal features below the sampling interval or below effective resolution after smoothing/downsampling; failing to report preprocessing that alters effective temporal resolution.
Model class/synchrony estimator	How synchrony is operationalized (e.g., contemporaneous covariation, lagged/linkage models, dynamical/coupled‐oscillator models; collapsed “overall synchrony” indices).	Specificity (primary); Generality (secondary); Sensitivity (secondary).	Determines which coordination structures are representable (e.g., simultaneous covariation, directional linkage, dynamical coupling), which features are estimable (e.g., sign, lag, asymmetry), and therefore which psychological interpretations are available to be tested. **Specificity:** It determines which coordination structures are estimable and therefore which interpersonal processes remain plausible. **Generality:** It conditions which comparisons across tasks, relationships, and timescales are warranted, given that different model classes operationalize coordination differently. **Sensitivity:** Model class constrains what dynamics can be detected at the chosen sampling rate/window.	**Interpretive:** Treating estimates from different model classes as interchangeable evidence for the same psychological claim; inferring mechanisms (e.g., transmission) from model classes that do not represent them. **Methodological:** Selecting a model class because it is customary or convenient rather than theory‐appropriate; comparing “synchrony” across estimators that define the construct differently; collapsing time‐varying coordination into a summary score that discards phase‐ or time‐localized structure needed for specificity.
Physiological target/index (HR vs. RSA vs. PEP, etc.)	Which system/branch is measured; single versus multimodal; composite versus branch‐specific.	Specificity (primary); Sensitivity (secondary).	Reduces physiological ambiguity by clarifying which regulatory influences could plausibly produce observed changes. **Specificity:** Branch‐specific and multimodal measurement can sharpen inference by supporting theory‐specified physiological profiles and reducing many‐to‐one ambiguity in single indices. **Sensitivity:** Measurement quality and artifact susceptibility affect detectability and the stability of synchrony estimates.	**Interpretive:** Treating global indices (e.g., HR) as diagnostically specific, or assuming indices are interchangeable and map one‐to‐one onto psychological constructs. In particular, misreading many‐to‐one mappings (multiple physiological pathways can produce the same observed index) as one‐to‐one evidence for a specific psychological process without convergent measures or theory‐specified profiles. **Methodological:** Poor signal quality or artifact handling (movement, ectopy, respiration confounding for RSA) that changes detectability and inflates apparent synchrony; failing to report index‐specific preprocessing choices.
Activation context/reference state (level + change)	Whether synchrony occurs at baseline, during reactivity, during recovery, or during change from another theoretically meaningful physiological state. Baseline is the most common reference point, but in some designs the relevant comparison may be a prior stressor, a preceding interaction epoch, or a previously identified autonomic configuration.	Specificity (primary); Generality (secondary); Sensitivity (secondary, when detectability of change is at issue).	Qualifies interpretation by situating synchrony within the level or state of physiological activation in which it occurs and, when relevant, by indexing each partner's magnitude and direction of deviation from a reference point. The same synchrony magnitude may have different implications when partners are coordinated around similar activation states versus when they fluctuate together while occupying different reference‐relative states, such as one partner above baseline and the other below. **Specificity:** Activation context can make activation‐dependent accounts (e.g., shared stress, threat, effort, recovery) more or less plausible, even though marked reactivity is not an eligibility condition for socially predictive synchrony. **Generality:** Baseline levels, recovery trajectories, and typical reactivity ranges vary systematically across contexts, populations, and measurement conditions, so the same synchrony pattern may not carry identical implications across samples or tasks. **Sensitivity:** Detecting activation context depends on design and measurement decisions, including how the reference state is defined, sampled, windowed, and preprocessed.	**Interpretive:** Conflating synchrony with arousal; assuming equal synchrony implies the same physiological or psychological state; ignoring direction of change relative to the relevant reference point; treating marked reactivity as required for socially meaningful synchrony. **Methodological:** Not measuring or inconsistently defining the relevant reference state, typically baseline; failing to indicate whether synchrony is estimated during baseline, reactivity, recovery, or another theoretically meaningful epoch; neglecting covariates that shift activation or reactivity, such as posture, respiration/speaking, or movement.
Context/task/relationship structure	Interaction type, motivational relevance, relationship type, status structure, etc.	Generality (primary); Specificity (secondary).	Defines boundary conditions for when an interpretation is warranted. **Generality:** Specifies where, when, and for whom a given synchrony interpretation is expected to replicate across tasks, relationships, populations, measurement settings, and timescales. **Specificity:** Contextual affordances and relational structure help distinguish among candidate psychological accounts that could produce similar observed synchrony (e.g., shared engagement vs. influence vs. common‐input structure).	**Interpretive:** Overgeneralizing interpretations across contexts with different affordances, demands, or relational asymmetries. **Methodological:** Underspecifying or failing to measure key contextual/structural features (task demands, role structure, shared pacing/stimuli) needed to adjudicate among candidate accounts.

*Note:* No single physiological synchrony feature is sufficient, by itself, to establish a specific psychological interpretation. Stronger inference typically requires joint consideration of specificity, generality, and sensitivity: whether the observed pattern and measurement profile narrow the range of plausible psychological interpretations; whether the interpretation is warranted under the relevant contexts, relationships, populations, and measurement conditions; and whether the chosen measures, sampling rate, windowing decisions, and model class can detect the dynamics specified by theory.

In brief, *magnitude* indexes the strength of synchrony between partners' signals; by itself it does not specify activation level or psychological salience. *Sign* distinguishes in‐phase synchrony, partners' signals moving together, from anti‐phase synchrony, in which they move in opposition, but its psychological meaning is underdetermined without theory about the interpersonal processes or contexts that would give rise to either pattern. *Directionality* and *lag structure* capture temporal organization and can constrain specificity by supporting some interpersonal processes (e.g., influence, contagion) while rendering others less plausible. *Observation window* and *sampling rate* primarily implicate sensitivity by determining which dynamics can be detected, if at all.

These formal features do not operate in isolation. Their psychological meaning depends on the physiological system in which synchrony is estimated, the activation context in which it occurs, and the broader interpersonal or theoretical context that gives synchrony its candidate meaning. Generality is strengthened across repeated studies when an inferred psychological meaning replicates across plausible methodological contexts, including relationship types, interaction paradigms, populations, and measurement settings and, where theory predicts, across related physiological indices. It is further strengthened when, within a theory‐appropriate model class, the inference remains robust to reasonable analytic specifications, such as window lengths, preprocessing choices, lag bounds, or parameter settings that do not alter the implied dynamics. Psychological interpretations are therefore strongest when these formal synchrony features and interpretive qualifiers are interpreted jointly, with explicit attention to the inferential constraints they satisfy: specificity, generality, and sensitivity. The next section considers how model class shapes which synchrony features and constraints can be represented analytically.

### 9.1 Model Class as an Inferential Constraint

Because formal synchrony features can only be interpreted through the analytic approach used to estimate them, the next inferential constraint concerns model class. Model class is not a neutral analytic choice, but an inferential constraint that shapes specificity, generality, and sensitivity at the same time: specificity by determining which coordination structures and psychological processes remain plausible; generality by defining the analytic conditions under which an inference can be replicated or shown to be robust; and sensitivity by determining which forms of physiological synchrony can be detected and represented. Different model classes emphasize different synchrony features and vary in whether they can preserve theoretically relevant distinctions, such as activation context, lag structure, directionality, or meaningful observation windows (key terms are defined in Table 7). In this sense, model choice shapes not only how synchrony is estimated, but which psychological interpretations are warranted.

**TABLE 7:** Model class and time‐series: key terms and phrases in plain language.

Key terms/phrases	Plain language
Model class	A family of analytic approaches that operationalizes physiological synchrony in a particular way, such as covariation, lead–lag prediction, coupling, or recurrent structure, and therefore constrains which interpersonal dynamics the analysis can represent.
Covariation	Partners' physiological signals rise and fall together (or in opposition) over time, typically measured as correlation or regression, usually without implying who influences whom.
Linkage	Changes in one person's signal are statistically associated with later changes in the partner's signal (i.e., a lead–lag connection), which can be consistent with interpersonal influence. In dyads, linkage may be unidirectional or bidirectional; in groups, it can be estimated across multiple directed connections.
Granger(−style) prediction	A prediction test in time series: one person's past values improve prediction of the partner's future values beyond what the partner's own past predicts. This supports directional predictability, not necessarily true causal influence.
State‐space formulations	Models that treat the signals as evolving over time through underlying (often unobserved) states, allowing synchrony to be represented as coordination in these latent dynamics rather than only in observed fluctuations.
Dynamical systems/coupled oscillator models	Models that represent each person's physiology as a dynamic system and estimate whether the partners' systems are coupled—that is, whether they mutually adjust over time in ways that may align with hypotheses about co‐regulation and/or co‐dysregulation (i.e., coordinated instability).
Recurrence‐based summaries	Approaches that quantify coordination by how often and how similarly partners' signals revisit the same patterns or states over time, rather than by correlation or prediction alone.
Global synchrony summaries	Approaches that reduce coordination across multiple signals, measures, dimensions, windows, or states into a single index of overall synchrony or linkage. These indices can be useful for summarizing broad physiological coordination, but they may collapse distinctions among physiological systems, sign, phase, lag, directionality, or time‐localized structure that are needed for more specific psychological inference.

*Note:* These terms refer to different operationalizations of physiological synchrony. Each model class emphasizes different features of coordination, such as covariation, lead–lag prediction, coupling, stability, recurrent structure, or overall coordination strength. The same dataset can therefore yield different synchrony estimates and warrant different psychological interpretations depending on the model class chosen.

Some approaches treat synchrony as contemporaneous covariation, such as correlations or regression‐based coupling (Helm et al. 2018; Marzoratti and Evans 2022). These approaches can estimate shared fluctuation and are well‐suited to questions about whether partners' signals covary within the same observation window, but they do not encode temporal ordering. Other approaches explicitly model temporal dependence, such as lagged coupling (Kraus and Mendes 2014; Liu et al. 2018), Granger‐style prediction (Fu et al. 2021; Granger 1969), or state‐space formulations (Gates and Liu 2016; Lodewyckx et al. 2011; Mildiner Moraga and Aarts 2024). These approaches can make inferences about influence or transmission more plausible by requiring temporal ordering, provided the design, sampling rate, and timescale support such claims. Still others model physiological synchrony as coupled systems (e.g., coupled oscillator/dynamical systems models), which can better align with hypotheses about co‐regulation or co‐dysregulation by estimating parameters such as attraction, damping, and stability (Boyd et al. 2022; Butler and Barnard 2019; Ferrer and Helm 2013). Recurrence‐based approaches represent another family of models, quantifying coordination by the extent to which partners' signals revisit similar states or patterns over time. These approaches may be useful for capturing recurrent structure, including multidimensional or group‐level dynamics (Wallot et al. 2016). However, when recurrence‐based analyses are summarized over an entire interaction or analysis window, the resulting summaries—such as recurrence rate, determinism, laminarity, or average diagonal length—can collapse distinctions that may be essential for specificity, including timing, phase, directionality, activation context, and, when multiple physiological systems or indices are modeled together, which physiological component or autonomic branch contributed most to recurrence. A related limitation arises in pipelines that reduce time‐varying coordination to global summary measures, such as “overall synchrony” indices computed across an entire interaction or task. Such summaries may improve comparability across studies but can obscure when coordination occurs, how it unfolds, and whether it is phase‐specific—information that may be essential for specificity (Gernert et al. 2024; Helm et al. 2018; Marci et al. 2007).

In short, modeling choices are not merely statistical conveniences: they are designed to represent different forms of synchrony and therefore align with different inferential aims. Model selection shapes specificity because the features represented by the model—such as simultaneity, sign, lag, directionality, stability, or recurrent state structure—constrain which interpersonal psychological processes remain plausible. It shapes generality because synchrony effects defined by different model classes may not be directly comparable: contemporaneous covariation, directional linkage, coupled dynamics, and recurrent state structure do not necessarily support the same psychological interpretation. And model class shapes sensitivity because each model class makes some forms of coordination detectable while leaving others unrepresented. Model selection should therefore be justified in relation to the psychological process and timescale under study. Models that incorporate temporal lags are better aligned with hypotheses about influence or transmission, whereas models that summarize simultaneous covariation are better aligned with hypotheses about shared engagement, concurrent coordination, or common‐input structure. When theories imply frequency‐specific coordination, recurrent structure, or dynamic state changes, model classes that preserve those features may be required; collapsing coordination into a single “overall synchrony” score can obscure these distinctions. This point is consistent with broader comparative work on pairwise time‐series interactions, which shows that analytic approaches differ in the kinds of dependencies they quantify, including contemporaneous, lagged, frequency‐specific, directionally predictive, and dynamically misaligned interactions (Cliff et al. 2023). Thus, choosing a simultaneous, lagged, state‐based, or dynamical operationalization is not merely a statistical preference; it is an inferential commitment about how the relevant interpersonal process is expected to unfold.

## 10 A Hypothetical Example Using Sympathetic Synchrony

To illustrate how these inferential constraints operate in practice, we use sympathetic nervous system (SNS) synchrony as a worked example. SNS reactivity is broadly associated with mobilization and arousal, but it is not psychologically specific: similar patterns of sympathetic activation can reflect stress, threat, excitement, or novelty (Kelsey 2012; Kelsey et al. 2000; Kreibig 2010). From the perspective of psychophysiological inference, SNS synchrony therefore cannot be assumed to carry a fixed psychological meaning independent of context and additional converging evidence. As noted, a further complication is that what qualifies as “synchrony” depends on how physiological synchrony is modeled. These modeling choices shape which synchrony features are defined and which interpersonal processes remain plausible candidates for interpretation—particularly with respect to whether observed synchrony is better explained by common inputs or by interpersonal influence or coordination on an interpretable timescale.

Imagine a study in which the same partners complete two interaction tasks designed to elicit sympathetic activation for different reasons. In the conflict task, partners discuss an unresolved disagreement (evaluative, antagonistic, high stakes). In the affiliative novelty task, partners complete a cooperative socially engaging challenge (novel, approach‐oriented). In both tasks, sympathetic activation is expected, and both tasks may show synchrony—yet the inferential question is what kind of synchrony appears and what alternatives remain plausible.

In the conflict task, one plausible interpersonal process is stress transmission. This process implies not merely that partners' sympathetic activity rises and falls together, but that changes in one partner's sympathetic activity are followed by similar changes in the other on an interpretable timescale, with additional corroborating evidence confirming that these changes reflect stress transmission rather than parallel responses to a common input. Accordingly, evidence for this account would include reliable time‐lagged synchrony—sometimes termed physiological linkage—especially if one partner's sympathetic shifts predict the other partner's subsequent shifts during identifiable escalation moments (e.g., criticism, defensiveness) and predict negative outcomes (e.g., dysregulation, social withdrawal, lower satisfaction, perceived stress). In the affiliative novelty task, similarly lagged coupling could support a different interpretation: one partner's excitement, amusement, or enthusiasm may precede similar changes in the other, reflecting the transmission of positive affect or approach‐oriented motivation rather than stress. Such an interpretation would be strengthened if lagged effects occurred around affiliative moments and predicted positive outcomes such as rapport, perceived similarity, willingness to affiliate, perceived novelty, or positive affect. Importantly, contemporaneous sympathetic covariation could also arise in either context but would not necessarily imply the same interpersonal process. In conflict, it might reflect joint engagement with an escalating stressor; in an affiliative task, it might reflect coordinated engagement, shared excitement, or mutual responsiveness. Thus, neither the presence nor timing of synchrony determines its psychological meaning on its own; interpretation depends on the task context, temporal scale, behavioral evidence, modeling approach, and associated outcomes.

Having specified these competing interpretations, researchers must also determine whether the observed synchrony reflects interpersonal influence at all. Superficially similar “sympathetic synchrony” could reflect common‐input structure rather than interpersonal influence in either task (e.g., turn‐taking, speech rate, task pacing). One straightforward design refinement is therefore to pair physiological synchrony estimates with time‐resolved behavioral markers (e.g., speaking turns, vocal intensity, shared event markers) and/or random dyads—that is, recomputing synchrony after pairing individuals with non‐partners (Danyluck and Page‐Gould 2019; Helm et al. 2012)—to test whether observed synchrony exceeds what would be expected from shared task structure or environmental drivers. If synchrony exceeds these common‐input structures, interpretation still hinges on whether the measurement and analysis can resolve the timescale implied by the hypothesized interpersonal process; that is, whether sampling rate and windowing permit detection of the relevant lag or dynamics. Depending on the hypothesized process, this logic may point to lagged dynamics, contemporaneous covariation, or both; what matters is whether the analytic timescale matches the interpersonal process being inferred.

From an inferential standpoint, these possibilities illustrate the same underlying principle. Without additional constraints, SNS synchrony is best treated as a psychophysiological concomitant: a broadly applicable physiological pattern that may be associated with a range of psychological processes but does not uniquely identify any one of them. Its psychological interpretation therefore depends on contextual constraints, task demands, temporal dynamics, and convergent behavioral or experiential evidence. Indeed, existing findings differ not only in context but also in the form of synchrony emphasized (e.g., lagged coupling during conflict versus contemporaneous covariation during affiliative interaction) and therefore in the inferential constraints they satisfy. For example, early work on marital conflict showed that dissatisfied couples exhibited multivariate physiological linkage during arguments—computed across several sympathetic sensitive measures—whereas satisfied couples did not (Levenson and Gottman 1983). In contrast, more recent work has shown that sympathetic synchrony between previously unacquainted partners during affiliative interactions predicts prosocial outcomes such as interest in friendship and perceived similarity (Danyluck and Page‐Gould 2018, 2019).

Because these findings differ substantially in relationship context, task demands, temporal modeling, and physiological operationalization—and thus in the inferential constraints they satisfy—they should not be treated as directly comparable at a methodological level. We raise them here not as competing tests of the same hypothesis, but as illustrative examples of how SNS synchrony has been interpreted differently across contexts. From the perspective of psychophysiological inference, such divergence is expected: SNS synchrony may carry distinct psychological meanings when the basis for interpretation differs.

This variability highlights a practical implication: contextual constraint is necessary but not sufficient for interpreting SNS synchrony. Specifying the interaction context helps define when a given interpretation is warranted and can narrow the range of plausible psychological accounts, but observations of SNS synchrony may still carry different meanings across constrained contexts. As illustrated earlier, physiologically aligned partners may not always be psychologically aligned, and context alone cannot determine whether synchrony reflects shared activation, interpersonal influence, common‐input structure, or a more specific psychological process. Stronger specificity therefore often requires multiple constraints to work together, including theory‐specified physiological profiles, temporally appropriate model classes, behavioral or experiential evidence, and activation contexts that distinguish among competing psychological interpretations.

The BPS model offers a useful test case for translating this logic to physiological synchrony in evaluative contexts: sympathetic activation is compatible with multiple psychological states, but synchrony embedded within a coordinated, baseline‐referenced cardiovascular profile—relatively higher cardiac output (CO) and lower total peripheral resistance (TPR) for challenge, or relatively lower CO and higher TPR for threat—could support more specific inferences about shared challenge or shared threat (Blascovich et al. 2003). Embedding synchrony within such discriminative physiological profiles could clarify what psychological accounts remain plausible and specify the boundary conditions under which the inference is warranted.

Yet this increased specificity comes with a corresponding increase in methodological burden. The BPS model is useful here not because it resolves the inferential challenges facing synchrony research, but because it makes those challenges especially visible. Although the model provides clear guidance for estimating challenge‐ and threat‐like profiles within individuals, these profiles are typically evaluated over aggregated task windows rather than tracked dynamically as people enter, maintain, and exit different cardiovascular configurations. Translating the BPS model to physiological synchrony therefore raises several challenges: whether both partners show sufficient engagement for BPS‐based interpretation, whether apparent coordination could reflect common inputs, and whether partners show comparable, mixed, or shifting challenge‐ and threat‐like profiles over time.

These timing issues matter because components of BPS profiles need not change in lockstep. Rapid vagal modulation can alter cardiac timing on a beat‐to‐beat basis, cardiac sympathetic influences on ventricular contractility and PEP reflect β‐adrenergic effects on the myocardium, and vascular and blood‐pressure components reflect baroreflex, hemodynamic, and sympathoadrenal processes with their own temporal properties (Di Rienzo et al. 2009; Fagraeus and Linnarsson 1976; Fontolliet et al. 2021; Goldstein 2010; Harris et al. 1967; Saul 1990). Consistent with this mechanistic logic, human acute‐stress and hemodynamic studies show that cardiac, vascular, sympathetic, and neuroendocrine components can vary across stressor type, repeated exposure, recovery phase, and individual differences (Al'Absi et al. 1997; Chen et al. 2024; Kelsey et al. 1999, 2000, 2004; Lackner et al. 2010; Obrist et al. 1974; Schächinger et al. 2001; Schneider et al. 2003).

Thus, before researchers can test whether partners synchronize challenge‐ or threat‐like profiles over time, they must first establish how such profiles can be validly identified dynamically within a person. Dyadic applications add further complexity: researchers must determine whether partners' profiles are temporally aligned with one another and with the interpersonal processes under study. These challenges are addressed in Box 3. The broader point is that theory‐specified physiological profiles can sharpen inference, but only when researchers can demonstrate that the profile is validly estimated, temporally aligned with the interpersonal process, and interpreted within clearly specified conditions under which the interpretation is warranted.

BOX 3 **Using BPS Challenge–Threat Profiles as a Test Case for Physiological Synchrony Inference.**

Before researchers can ask what it means for two people's challenge‐ or threat‐relevant profiles to become synchronized, they must establish that those profiles are interpretable within each person across the period over which synchrony is modeled. This does not mean that intrapersonal issues can be fully resolved before interpersonal ones arise. Rather, many of the same challenges—motivational engagement, temporal coherence among cardiovascular components, measurement sensitivity, habituation, and analytic windowing—operate within individuals and are compounded when two partners vary in these features across time. BPS‐relevant synchrony therefore requires attention to two linked questions: whether each person's profile can be validly estimated in the relevant window, and whether coordination between those profiles supports claims about shared challenge, shared threat, stress transmission, co‐regulation, or some other dyadic process.

**Motivational engagement and profile eligibility**. BPS profiles are interpretable only when the task is motivationally relevant and requires active coping. In a single person, this means that challenge‐ or threat‐relevant profiles should not be interpreted unless there is evidence of engagement during the window in which the profile is estimated. In dyadic synchrony research, this requirement is compounded because partners may differ in whether, when, and how strongly they are engaged. One partner may show clear engagement while the other shows little evidence of active coping; both may be engaged but occupy different positions on the challenge–threat continuum; or each partner may move along that continuum at different moments in the interaction. Experimental manipulations may therefore produce challenge‐like or threat‐like responses on average while still yielding substantial within‐person, between‐person, and dyad‐level heterogeneity. In addition, canonical BPS profiles may be easier to estimate and interpret in some participants than others because cardiovascular response patterns vary by age, baseline physiology, blood‐pressure status, cardiovascular risk, and vascular or baroreflex function (Di Rienzo et al. 2009; Duschek et al. 2008; Ghiadoni et al. 2000; Schneider et al. 2003; Uchino et al. 1999). Cardioactive medication use and other hemodynamic factors may also affect estimates of PEP, CO, and TPR, and these physiological differences can be compounded by measurement sensitivity when profiles are estimated dynamically or compared across partners (Harris et al. 1967; Krohova et al. 2017; MacCormack et al. 2021). Researchers interested in BPS‐relevant synchrony therefore need to describe not only whether partners are synchronized but whether the profiles being synchronized are interpretable for each person in the relevant window.

**Temporal coherence and profile dynamics**. A second challenge is whether the components of the BPS profile can be estimated as a temporally coherent pattern. Within a person, PEP, CO, and TPR may not emerge, habituate, or recover on the same timescale. Cardiac, vascular, sympathetic, and hemodynamic components can show dissociable onset, adaptation, and recovery patterns across acute stress, repeated exposure, and recovery (Kelsey et al. 1999, 2000, 2004; Lackner et al. 2010; Obrist et al. 1974; Schächinger et al. 2001; Schneider et al. 2003). If these components are aggregated over mismatched windows, the resulting profile may not represent a single psychophysiological state. In dyadic synchrony research, this problem is compounded because partners may shift between more challenge‐like and more threat‐like profiles at different times. As demands, resources, feedback, appraisals, or partner behavior change, synchrony estimates may therefore combine periods in which partners are not occupying comparable motivational or physiological states. This is especially problematic when some profile components can be estimated continuously from ECG/ICG‐derived measures whereas others, such as TPR, depend on blood pressure that may be measured intermittently. Continuous blood pressure measurement may improve temporal alignment, but it introduces practical challenges, including cost, participant burden, motion artifact, and possible incompatibility with naturalistic interpersonal tasks.

**Design implication**. BPS‐relevant synchrony studies should be designed around these constraints from the outset. Rather than averaging over long, heterogeneous interactions, researchers may need repeated, brief motivated‐performance episodes that preserve uncertainty or evaluation, permit temporally aligned measurement of cardiovascular components, and allow shared task events to be modeled directly. State‐space or related state‐switching models (Lodewyckx et al. 2011; Mildiner Moraga and Aarts 2024; Pohle et al. 2021) may be useful in this context because they can, in principle, represent challenge‐ and threat‐relevant physiology as evolving states rather than as fixed profiles averaged across an entire interaction; however, applying these approaches to BPS‐relevant synchrony would require additional methodological work to establish whether inferred states correspond to theoretically specified challenge‐ and threat‐like profiles and whether transitions among partners' states can be interpreted as meaningful psychological or interpersonal dynamics. Doing so would require clear a priori theory, dense and temporally aligned measurement, sufficient observations within states, careful validation of the inferred states, and resolution of data‐analytic challenges that may arise when modeling multiple physiological streams from two or more people simultaneously. The goal is not simply to show that partners' profiles covary, but to establish that each person's profile is interpretable, that the profile components refer to the same psychophysiological window, and that coordination among profiles supports the psychological interpretation being advanced.

## 11 Design Principles for Stronger Inference

**FIGURE 3:**
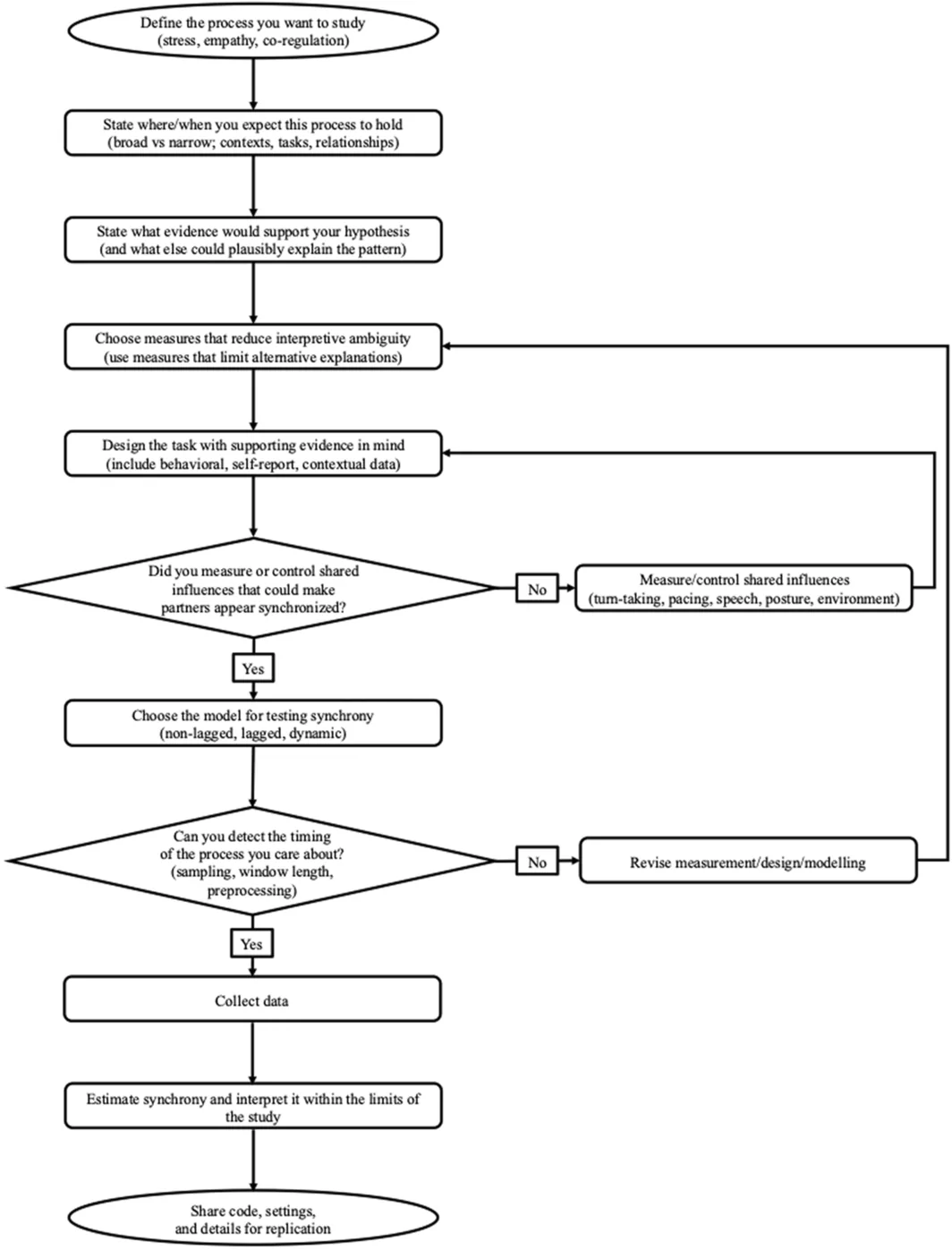
Workflow for psychophysiological inference in physiological synchrony research. This workflow operationalizes the design principles described in the text by expanding them into a sequence of 11 practical steps, including two diagnostic checkpoints (common inputs/shared influences; sensitivity/detectability) that prompt iteration to earlier steps when not satisfied. This workflow is intended as a guide for strengthening inference rather than a requirement that every study satisfy every step; studies can remain informative when they clearly specify which constraints are addressed, which remain unresolved, and how those limits bound interpretation. (1) Define the interpersonal psychological process of interest (e.g., stress transmission, empathy, co‐regulation). (2) Specify intended conditions of generality a priori (i.e., where/when the interpretation is expected to hold), including whether it is expected to generalize broadly across contexts, tasks, relationships, and timescales (context‐free) or only under specified conditions (context‐dependent). (3) Derive discriminative predictions and plausible alternatives (specificity), including expected temporal structure when theory implies transmission, influence, shared fluctuation, or other coupled dynamics. (4) Select measures to reduce physiological ambiguity, favoring more specific indices or theory‐specified profiles when global endpoints are insufficient for the intended claim. (5) Design the task with convergent evidence in mind and specify behavioral, self‐report, and contextual measures that can help distinguish among candidate psychological interpretations. (6) Anticipate common‐input structure and measure/control shared influences that could make partners appear synchronized (e.g., turn‐taking, pacing, speech/respiration, posture, environment); plan robustness checks (e.g., random dyads). (7) Choose a model class as an a priori analytic commitment that matches the theorized dynamics, such as contemporaneous covariation, lagged linkage, dynamic coupling, state‐space models, or recurrence‐based approaches. (8) Verify sensitivity/detectability for the theorized timing and structure of synchrony, including sampling rate, window length, preprocessing, and signal quality. (9) Collect data. (10) Estimate synchrony and interpret it only within the stated constraints, including the intended conditions of generality and any satisfied checkpoints. (11) Support reproducibility and replicability by sharing analysis code, settings/specifications, and reporting decisions in sufficient detail to enable direct replication in new samples. Although shown sequentially for clarity, task design and model choice are often developed iteratively: Anticipated synchrony dynamics can shape task design, and design constraints can in turn limit which models are appropriate.

Taken together, the preceding sections and worked examples suggest several design principles for strengthening psychophysiological inference in physiological synchrony research (see Figure 3 for an expanded workflow that operationalizes these principles).^6^ We do not intend these considerations to imply that every study must test all possible features of synchrony, include multiple physiological systems, or establish generality across methodological contexts in a single design. In practice, physiological synchrony research is constrained by available equipment, participant burden, sample size, task structure, signal quality, and analytic feasibility. The goal is therefore twofold: to guide researchers toward designs that strengthen psychological inference by building in stronger theoretical, physiological, contextual, and analytic constraints, and to help researchers calibrate their claims when practical limitations prevent all constraints from being addressed in a single study.

First, physiological synchrony should be treated as an empirical pattern that requires interpretation, not as a psychological construct in its own right. Second, physiological measures should be selected to minimize physiological ambiguity, favoring branch‐specific indices and/or theory‐specified physiological profiles when claims hinge on specific rather than broad psychological or interpersonal processes. Third, when activation‐based interpretations are intended, study designs should deliberately elicit physiological activation for theoretically distinct psychological reasons—such as evaluative threat versus affiliative novelty—and pair synchrony estimates with convergent behavioral, experiential, and contextual data to distinguish among candidate psychological interpretations. Fourth, interpretations should remain explicitly bounded: scientists should clearly articulate the intended conditions of generality—for example, the populations, relationships, tasks, and timescales over which an interpretation is expected to hold—and the boundary conditions under which it should not be assumed. Fifth, formal models of physiological synchrony should be treated as inferential commitments: choices about sampling rate, timescale, observation window, and model class constrain what dynamics are detectable (sensitivity), which psychological interpretations remain plausible (specificity), and the conditions under which those interpretations are warranted (generality). Sixth, because synchrony estimates can be highly contingent on preprocessing and modeling decisions, authors should support computational reproducibility and replicability by sharing research protocols, preprocessing and modeling pipelines, and analysis code, and by reporting analytic decisions in sufficient detail to enable direct replication in new samples. Together, these principles reorient physiological synchrony research away from demonstrating that physiological synchrony occurs and toward clarifying which psychological interpretations are warranted, under what conditions, and with what inferential constraints.

When these recommendations are implemented partially, studies can still make important contributions by making clear which inferential constraints are addressed, which remain unresolved, and how those limits bound interpretation. For example, a study that models synchrony in a single physiological index during one interaction context may provide evidence for a phenomenon under those specific conditions but should avoid treating that pattern as evidence for a general psychological process unless additional theoretical, contextual, behavioral, or physiological constraints support that inference. Progress in this area will therefore often be cumulative: individual studies can test whether synchrony occurs under theoretically meaningful conditions, while programs of research can evaluate whether the same pattern generalizes across contexts, measures, populations, and analytic specifications. More definitive progress on the generality of physiological synchrony may ultimately require coordinated multi‐site efforts using standardized tasks, harmonized measurement protocols, and transparent analytic pipelines. Such designs would allow the field to test whether particular synchrony patterns support similar psychological interpretations across populations, relationships, contexts, and model specifications.

## 12 Implications for Contemporary Theories of Physiological Synchrony

The present framework is intended to complement, rather than replace, contemporary theories of physiological synchrony. Existing models have made substantial progress in explaining how physiological synchrony emerges, how it changes over time, and why it may matter for social development, affiliation, co‐regulation/co‐dysregulation, and affect transmission (Feldman 2012; Gordon et al. 2024; West and Mendes 2023). The contribution of the present framework is different: it specifies the inferential steps by which particular physiological synchrony estimates can support particular psychological interpretations. We illustrate this complementarity by applying the framework to three perspectives that have shaped physiological synchrony research: the biobehavioral model of synchrony (Feldman 2012), the theory of flexible multimodal synchrony (Gordon et al. 2024; Gordon and Bartsch 2026), and the theory of affect contagion (West and Mendes 2023).

### 12.1 Biobehavioral Model of Synchrony

The biobehavioral model of synchrony is a developmental theory of how repeated, temporally organized caregiver–infant coordination across behavior, physiology, hormones, and neural systems becomes embedded in affiliative bonds over time (Feldman 2012). Physiological synchrony is not the model's sole focus; rather, it is one expression of a broader biobehavioral system linking social timing, biological organization, caregiving context, and regulatory development. This breadth is a strength because it situates physiological synchrony within microcoded behavior, developmental timing, caregiving variation, and longitudinal outcomes.

The present framework builds on this strength by asking what additional constraints are needed when physiological synchrony is interpreted as evidence for a specific process such as co‐regulation, external regulation, resilience, or stress transmission. For example, mother–infant heart rhythm coupling during coordinated gaze, vocalization, and affect may be consistent with co‐regulation or external regulation, but stronger inference would require evidence that coupling is most pronounced when regulation is needed, that caregiver behavior or physiology predicts subsequent adaptive change in the infant, that the physiological signal indexes recovery or flexible engagement rather than nonspecific arousal matching, and that the pattern predicts later stress‐regulatory competence beyond behavioral synchrony alone. Without such constraints, similar coupling could also reflect shared attention, coordinated movement, touch‐mediated physiological stabilization, parallel arousal, common sensory input, or infant entrainment of the caregiver.

This point is not meant to diminish the biobehavioral approach. On the contrary, this research program already contains many elements that can strengthen inference when physiological synchrony is measured directly. Microcoded behavior can support specificity by identifying the behavioral channels through which physiological coupling may arise. Developmental timing, caregiving variation, and risk contexts can support generality by specifying when, for whom, and under what relational conditions a given interpretation is warranted. Longitudinal outcomes can help evaluate whether a synchrony pattern predicts the developmental endpoints assigned to it by theory. However, prediction and explanation place different demands on evidence. Physiological synchrony may serve as a useful developmental or prognostic marker even when its mechanism is underspecified, but once it is invoked as a mechanism of external regulation, optimal parenting, resilience, or intervention, its psychological and physiological interpretation becomes central.

### 12.2 The Theory of Flexible Multimodal Synchrony

The theory of flexible multimodal synchrony emphasizes that synchrony is dynamic, multimodal, metastable, and context‐sensitive, drawing attention to the fact that social systems may move into and out of synchrony over time and that desynchronization may itself be meaningful (Gordon et al. 2024; Gordon and Bartsch 2026). This perspective aligns closely with the present framework's emphasis on generality and sensitivity: synchrony should not be interpreted as a static property of an interaction, nor should average coupling across an entire task be assumed to capture the dynamics most relevant to psychological interpretation.

The present framework further complements this theory by shifting the starting point of synchrony research from synchrony itself to the interpersonal psychological process that makes synchrony theoretically meaningful. Terms such as “pulls toward synchrony and segregation” are useful for describing dynamic tendencies in social systems, but they do not by themselves specify the psychological or psychophysiological conditions under which a particular synchrony pattern should emerge. People are not typically motivated to synchronize their physiology; rather, they may be motivated to connect, soothe, persuade, monitor, avoid, or coordinate action. Physiological synchrony may then emerge as a downstream consequence of these interpersonal orientations, task affordances, and available channels of information exchange. Applied to the theory of flexible multimodal synchrony, the perspective advanced here shifts the relevant question from whether a context pulls toward synchrony or segregation in general to what interpersonal process would make one partner's state or behavior relevant to the other in ways that could give rise to physiological synchrony. That is, the present framework encourages attention to why social partners might track, anticipate, or respond to one another in a given context; what contextual conditions would enable or amplify that process; and what pattern of physiological, neural, or behavioral coordination, temporal structure, and boundary conditions would be required to support that interpretation. Contexts can create affordances that make synchrony or desynchrony more likely, but those affordances do not by themselves specify the psychological process or warrant the interpretation.

This reframing also clarifies the role of context. Contextual interpretation can become circular when researchers infer cooperation, connection, or empathy merely because synchrony occurred during a cooperative, affiliative, or empathic task (Gordon and Bartsch 2026). However, context should not be treated only as a source of interpretive bias. In psychophysiological inference, context is part of the evidentiary basis for interpretation because physiological signals are not psychologically self‐interpreting (Cacioppo and Tassinary 1990). The problem is not using context; the problem is using context circularly. Context should therefore be specified a priori, experimentally varied or independently validated where possible, and used to generate discriminative predictions. Circularity is reduced when the same physiological pattern is tested across theoretically contrasting contexts, behavioral states, and outcome measures, and when researchers can show that the findings are more consistent with the proposed interpersonal process than with plausible alternative explanations.

The theory's emphasis on flexibility and metastability also provides a useful bridge to autonomic modes of control. From an autonomic‐space perspective, movement into and out of synchrony may reflect changes in how individuals coordinate sympathetic and parasympathetic activity to meet shifting social and self‐regulatory demands. At some moments, aligning with another person's physiology may serve an individual's goals, such as when a caregiver tracks an infant's state, teammates coordinate action, or a person monitors another for threat. At other moments, the individual may prioritize recovery, disengagement, independent action, emotion regulation, or resistance to another person's affective state. Thus, flexible synchrony may reflect not only movement between social connection and segregation but also dynamic reorganization of autonomic control as social demands become more or less relevant to the individual's regulatory priorities.

A key contribution of the flexible multimodal synchrony account is that it reframes synchrony as a flexible, context‐sensitive dynamic rather than as an inherently adaptive or maladaptive outcome. From this perspective, the adaptiveness of synchrony depends less on its absolute magnitude than on whether dyads can enter, maintain, exit, and reorganize synchrony in ways that fit the current psychological and physiological demands of the interaction. The present framework complements this account by clarifying how such claims can be evaluated when stronger psychological inference is the goal. Such inference is supported when researchers can specify which psychological process is implicated by a particular synchrony dynamic, identify the conditions under which synchrony or desynchrony is expected to be adaptive, and use designs and models sensitive to the temporal scale on which that dynamic unfolds. Multimodal evidence strengthens inference not simply by adding more channels, but by linking each channel to discriminative predictions about which modality should synchronize, in what direction, at what lag, under which conditions, and relative to which alternative explanations. Continuous synchrony may be adaptive during infant soothing, musical performance, or coordinated movement, but maladaptive during conflict escalation, co‐rumination, or tasks requiring independent creativity. Likewise, desynchrony may be adaptive when autonomy, recovery, or complementary role performance is needed, but maladaptive when attunement, repair, or co‐regulation is required. In this way, the theory of flexible multimodal synchrony identifies dynamic phenomena that synchrony researchers should study, whereas the present framework specifies the inferential conditions under which those dynamics can support particular psychological interpretations.

### 12.3 The Theory of Affect Contagion

The theory of affect contagion provides a particularly useful example of how physiological synchrony can be situated within a defined interpersonal psychological process (West and Mendes 2023). Rather than treating synchrony as the focal construct, this framework begins with affect contagion: the transmission and detection of affective states from one person to another. This emphasis is valuable because it positions physiological synchrony as evidence relevant to a specified interpersonal process, rather than as a psychological process in itself.

The theory also illustrates a key principle of the present framework: psychological claims should be scaled to the specificity of the physiological evidence. Affect contagion is a psychologically meaningful but relatively broad target. It concerns the transmission of affective states, not necessarily the sharing of discrete emotions such as sadness, anger, fear, or joy. Physiological measures used to study affect contagion can therefore be calibrated to that level of inference. For example, PEP need not be treated as an index of a discrete emotion; it can instead function as an index of activated affective responding, which is well matched to a broad theory of affect contagion. This logic is already exemplified by the study illustrated in Box 1. In that study, the target construct was affect transmission, the originating affective state was experimentally constrained and validated, and mother–infant physiological covariation was interpreted alongside manipulation checks, infant physiological reactivity, and infant behavioral evidence (Waters et al. 2014). The synchrony estimate was therefore not asked to carry the full weight of the psychological inference on its own.

This scaling logic is important because stronger claims about shared discrete emotions would require additional constraints on specificity, generality, and sensitivity. Given evidence against one‐to‐one mappings between discrete emotions and autonomic profiles (Siegel et al. 2018), such inferences would likely depend less on identifying a unique physiological signature than on triangulating physiological synchrony with context, behavior, self‐report, temporal dynamics, and theoretically relevant alternative explanations. In this sense, the theory of affect contagion illustrates a scalable form of specificity: broad constructs can be tested with broader physiological indicators when the context and convergent evidence constrain the interpretation, whereas narrower psychological claims require more discriminative evidence.

The process‐first logic of affect contagion also parallels the model‐selection principles advanced in the present framework. The theory identifies plausible low‐ and high‐level antecedents of affect contagion, including sensory cues, expressive behavior, mentalizing, partner readability, motivation, and status. It also distinguishes physiological covariation from physiological linkage, mapping different analytic models onto different interpersonal claims. Covariation is more consistent with shared affective response to a common situation, whereas linkage is better suited to claims about directional influence, in which one partner's prior physiological state predicts the other partner's subsequent physiological change. This distinction illustrates how analytic choices constrain psychological interpretation: model selection is not merely statistical, but theoretical.

The theory of affect contagion also highlights timing and activation context. Its emphasis on covariation versus linkage implies that analytic windows, lags, and model classes should be selected because they match the expected timing of the affective and physiological process under study, not simply because they are conventional or convenient. This becomes especially important when affect contagion is theorized to unfold through feedback loops, such that interpersonal accuracy increases affective coupling, which in turn increases later accuracy or regulatory attunement. Similarly, the theory identifies arousal as a key moderator and notes that sympathetic measures may be especially useful in activating contexts, whereas parasympathetic measures may be more appropriate for lower‐arousal states. The present framework formalizes this point by treating activation context not only as a condition for detecting synchrony but also as a constraint on its psychological interpretation. Comparable levels of synchrony may support different interpretations depending on whether they occur during high activation, such as conflict or threat, or during lower activation, such as calm affiliation, relaxation, or social engagement.

In short, the theory of affect contagion provides a useful demonstration of the interpretive clarity that can emerge when synchrony research begins with a defined interpersonal process. The theory applies inferential constraints in a relatively broad domain by defining the target process, identifying plausible antecedent channels, selecting physiological measures suited to activated affect, and distinguishing analytic models that correspond to different interpersonal claims. The present framework complements this account by making the underlying inferential logic explicit, linking it to classical principles of psychophysiological inference, and showing how similar logic can be scaled to narrower or alternative psychological targets. Broad constructs such as affect contagion may be tested with broader physiological indicators, whereas narrower claims about co‐regulation, empathy, affiliation, threat, dominance, resilience, joint action, or rupture‐repair require more discriminative evidence. In each case, stronger inference is supported when researchers can specify the interpersonal process, the contexts in which the interpretation is expected to hold, the physiological measures that match the intended claim, and the temporal structure needed to detect the hypothesized dynamics.

Taken together, these three perspectives show that contemporary theories of physiological synchrony already offer valuable accounts of when synchrony emerges, how it unfolds, and why it may matter. The biobehavioral model situates physiological synchrony within developmental systems of social timing, caregiving, biological organization, and long‐term adaptation. The theory of flexible multimodal synchrony highlights the dynamic, multimodal, metastable, and context‐sensitive nature of synchrony and desynchrony. The theory of affect contagion illustrates how synchrony can be embedded within a defined interpersonal process and linked to distinct forms of covariation and linkage. The present framework adds a complementary layer by specifying the inferential conditions under which observed physiological synchrony can support particular psychological interpretations.

## 13 Toward a Shared Inferential Framework for Physiological Synchrony Research

By foregrounding psychophysiological inference, the present framework clarifies the conditions under which physiological synchrony can be meaningfully linked to psychological processes. Physiological synchrony arising from co‐regulation, shared task engagement, affect contagion, or common environmental demands may be statistically indistinguishable at the level of observed coordination, yet psychologically distinct. The inferential constraints articulated here—specificity, generality, and sensitivity—provide a way to distinguish among these possibilities by asking what the observed physiological pattern rules in or out, where and when that interpretation is expected to hold, and whether the measures and models are capable of detecting the hypothesized interpersonal dynamics. Because model class determines which forms of coordination are representable and which synchrony features are defined, theoretical claims should be evaluated against the specific model used to estimate synchrony—not “synchrony” in the abstract.

By providing a shared inferential vocabulary for features of physiological synchrony, boundary conditions, and model class, the present framework supports cumulative interpretation across heterogeneous designs rather than isolated juxtapositions. These principles do not imply that psychologically meaningful inference requires synchrony in both autonomic branches. Temporally structured, branch‐specific synchrony can be informative when interpreted within explicit boundary conditions and triangulated with convergent evidence. At the same time, theory‐specified physiological profiles can sharpen specificity by clarifying which physiological pattern would support a given psychological interpretation.

A fuller integration of psychophysiological inference with specific dynamical theories of physiological synchrony represents an important direction for future work. Such efforts might examine how different models constrain the range of plausible psychological meanings associated with particular physiological synchrony patterns, or how inferential principles can guide the selection of measures, timescales, and contexts most appropriate for testing competing theoretical claims. The present contribution lays the groundwork for this integration by clarifying the inferential logic that should guide psychologically meaningful interpretation of physiological synchrony. The goal is not to assign a universal psychological meaning to synchrony, but to specify the inferential conditions under which particular meanings can—and cannot—be inferred.

## 14 Conclusion

Physiological synchrony has captured the imagination of psychological scientists because it offers a compelling vision of shared embodiment—of minds and bodies moving together in real time. Yet without a principled framework for psychophysiological inference, this promise risks outpacing its explanatory power. The present paper argues that physiological synchrony is not itself a psychological construct, but an empirical pattern whose meaning depends on inferential constraints grounded in specificity, generality, sensitivity, and theory. By extending classical principles of psychophysiological inference to the study of interpersonal physiology, we provide a foundation for interpreting physiological synchrony with greater precision and restraint. Importantly, this framework does not diminish the value of physiological synchrony as a phenomenon of interest. Rather, it clarifies what physiological synchrony can—and cannot—tell us about psychological processes, and under what conditions stronger inference is warranted. By shifting attention from whether physiological synchrony occurs to what it means and how to interpret it, the field can move beyond demonstrations of physiological synchrony toward a more cumulative and theoretically constrained literature on its psychological meaning.

## Author Contributions

**Chad Danyluck:** conceptualization, writing – original draft, visualization, writing – review and editing, supervision. **Andy Adler:** writing – original draft, writing – review and editing, conceptualization. **Tyler Thorne:** writing – original draft, writing – review and editing. **Nissa Doyle:** writing – original draft, writing – review and editing.

## Conflicts of Interest

The authors declare no conflicts of interest.

## Data Availability

Data sharing is not applicable to this article as no datasets were generated or analyzed during the current study.

## Endnotes

^1^
Although physiological synchrony can be studied at neural as well as peripheral levels, the present review focuses on peripheral psychophysiology (primarily autonomic and cardiovascular indices) because these measures dominate the empirical synchrony literature in psychology and raise distinctive inferential challenges (e.g., dual innervation and endpoint ambiguity). We return to implications for neural synchrony and multilevel integration toward the end of this review.

^2^
Readers seeking further background may consult the foundational and integrative work on psychophysiological inference (Cacioppo et al. 2016; Cacioppo and Tassinary 1990), autonomic determinism and the modes of autonomic control (Berntson et al. 1991), and overviews of psychophysiological recording methods and interpretation (Berntson et al. 1994, 1997; Blascovich et al. 2011; Cacioppo et al. 2017; Camm et al. 1996; Kelsey 2012; Kelsey et al. 2000; Porges 1995; Quigley et al. 2024; Sherwood et al. 1990).

^3^
This ordering is not meant to imply that generality is secondary. We address specificity first because it clarifies how physiological observations narrow the range of plausible psychological interpretations; we then turn to generality as a complementary constraint on when, where, and under what conditions those interpretations are warranted.

^4^
For readers unfamiliar with the autonomic modes of control framework, Berntson et al.'s (1991) original figures—particularly Figure 4—provide useful visual depictions of sympathetic and parasympathetic configurations within the autonomic space. Table 3 adapts this logic to the interpretation of physiological synchrony.

^5^
More formally, the time–frequency uncertainty principle (Cohen 2001; Gabor 1946) implies a lower bound on the product of temporal and spectral dispersion (e.g., *T*_*SP*_ *B*_*SP*_ ≥ 1). A window of duration, T, minutes cannot resolve frequency structure finer than 1/T cycles per minute. Thus, a 10‐min window yields frequency resolution on the order of 0.1 cycles/min, meaning attempts to isolate a “synchronous” rhythm near 1 cycle/min will inevitably admit nearby content between 0.95 and 1.05 cycles/min—limiting the maximum possible attenuation of non‐synchronous components within that epoch.

^6^
Parallel inferential constraints apply in neural (inter‐brain) synchrony: recorded signals can reflect mixtures of underlying generators, including volume conduction or field spread in EEG/MEG, and, in modalities such as fNIRS, systemic physiological contamination (Nolte et al. 2004; Schoffelen and Gross 2009; Vinck et al. 2011; Yücel et al. 2021). Apparent inter‐brain synchrony can also arise from common inputs, such as shared stimuli or interaction structure, rather than interpersonal synchrony per se (Burgess 2013). Interpretations are likewise sensitive to model‐class choices and temporal resolution, motivating leakage‐resistant metrics or source‐level approaches when appropriate (Schoffelen and Gross 2009; Vinck et al. 2011; Yücel et al. 2021; Zimmermann et al. 2024).
